# HDAC3-Regulated PGE2 Production by Microglia Induces Phobic Anxiety Susceptibility After Stroke and Pointedly Exploiting a Signal-Targeted Gamma Visual Stimulation New Therapy

**DOI:** 10.3389/fimmu.2022.845678

**Published:** 2022-02-18

**Authors:** Hongrui Zhu, Yi Guo, Ailing Huang, Huidan Shen, Yang Chen, Jingyi Song, Ao Guan, Liang Wu, Huiting Wang, Bin Deng

**Affiliations:** ^1^ Department of Anesthesiology, Xiang'an Hospital of Xiamen University, School of Medicine, Xiamen University, Xiamen, China; ^2^ Department of Anesthesiology, The First Affiliated Hospital of Xi'an Jiaotong University, Xi'an, China; ^3^ Department of Anesthesiology, First Affiliated Hospital of USTC, Division of Life Sciences and Medicine, University of Science and Technology of China, Hefei, China; ^4^ State Key Laboratory of Cellular Stress Biology, Xiamen University, Xiamen, China; ^5^ Department of Neurology, The 904th Hospital of PLA, Medical School of Anhui Medical University, Wuxi, China; ^6^ School of Medicine, Xiamen University, Xiamen, China

**Keywords:** poststroke anxiety, histone deacetylases, microglia, prostaglandin E_2_, amygdala, gamma oscillation

## Abstract

**Background:**

Phobic anxiety present after stroke (called poststroke anxiety, PSA) can hamper the rehabilitation of patients and disrupt their usual activities. Besides, the symptoms and mechanisms of PSA are different from those in nonstroke populations that have generalized anxiety disorder. What’s more, the treatment approaches for phobic anxiety are confined to unitary or general methods with poor efficiency.

**Methods:**

Behavioural test screen combined bioinformatics analysis explored molecular changes between generalized anxiety disorder in nonstroke mice (restraint stress, RS) and photothrombotic stroke mice exposed to environmental stress (PTS + RS, mimicking PSA). Multiple molecular biological and neurobiological methods were employed to explain mechanisms *in vitro* and *in vivo*. And exploiting gamma flicker stimulation device for therapy.

**Results:**

Microglial (MG) overactivation is a prominent characteristic of PTS + RS. HDAC3 was mainly upregulated in activated-microglia from damaged cortex and that local prostaglandin E2 (PGE_2_) production increased in MG *via* HDAC3-mediated activation of NF-κB signalling by p65 deacetylation. A high content of PGE_2_ in damaged ischaemic cortex could diffuse freely to amygdala, eliciting anxiety susceptibility of PSA *via* EP2. Importantly, gamma flicker stimulation relieved anxious behaviour of PTS + RS by modulating the HDAC3/Cox1/EP2 network at some extent.

**Conclusions:**

HDAC3-regulated PGE_2_ production by microglia constitutes phobic anxiety susceptibility after stroke and a protective approach of gamma visual stimulation can be a candidate new therapy.

**Graphical Abstract:**
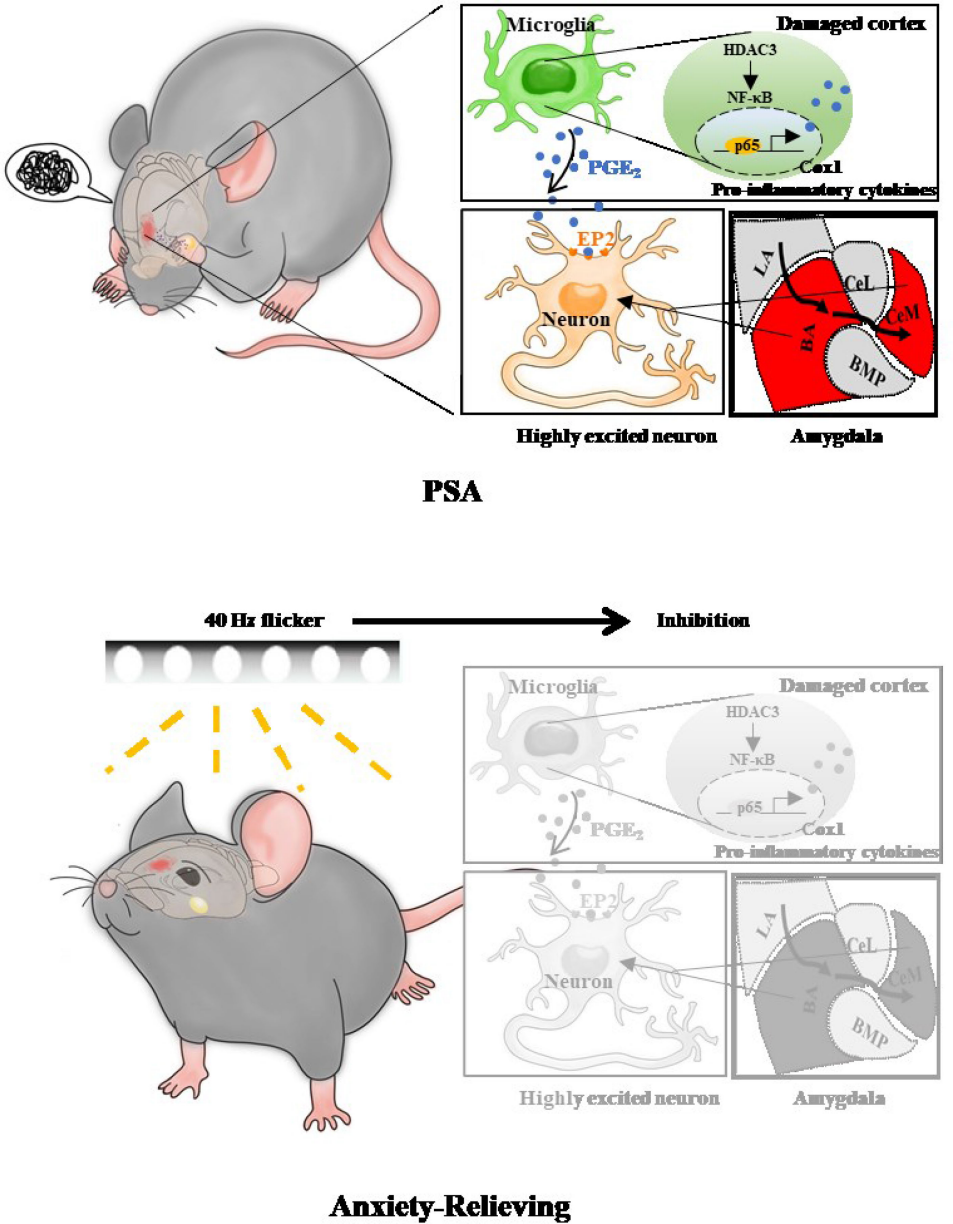


## Introduction

Poststroke anxiety (PSA) is common, emerging from a quarter of strokes and approximately a third of transient ischaemic attacks (TIAs) (1). Most evidence indicates that phobic anxiety present after stroke can hamper the rehabilitation of patients and disrupt their usual activities. Intervention approaches for phobic anxiety are limited to unitary or general methods, such as relaxation and anti-depressants, which are treatments for generalized anxiety and are unlikely to be effective in phobic anxiety (2, 3). Therefore, researchers recognize different pathophysiological changes in nonstroke populations that generalize anxiety disorder (GAD) and stroke patients with phobic anxiety. Populations with GAD usually manifest persistent and multiple worries, such as finance or health, while phobic anxiety represents unpleasant anxiety of situations or stimuli (3). Phobic disorder is the predominant anxiety subtype after stroke or TIA (4) and is markedly different from generalized anxiety (5). In addition, stroke individuals with young or previous anxious and depressed characteristics usually elicit the likelihood of phobic anxiety (2, 3). Pathologically, patients with right posterior lesions had more anxiety than those with right anterior lesions. Left cortical or subcortical anterior lesions increase individual vulnerability and decrease resilience under environmental stress, although areas of regulating negative emotion are not necessarily directly impaired (e.g., the greater ventral tegmental area (VTA), the nucleus accumbens (NAc), the prefrontal cortex (PFC) and the amygdala). However, pathophysiologically, little is known about these underlying molecules that contribute to resilient/vulnerable anxiety following stroke. Accordingly, identification of key molecules involved in anxiety susceptibility remains a priority.

Epigenetic remodelling factors have been reported to participate in anxiety susceptibility in chronic stress models (6). Recent evidence has shown that histone deacetylases (HDACs) are not only connected with emotion regulation, such as depression, but are also involved in poststroke recovery (7, 8). For example, HDAC3 and HDAC6 manifest differential expression levels in chronic social defeat stress (CSDS) mice. HDAC3, mediating deacetylating histones or non-histones, is involved in enhancing the tolerance of cerebral hypoperfusion and ischaemia/reperfusion and upregulating in damaged brain tissue or activated microglia (9, 10). In addition, HDAC3 limits the inflammatory reaction in immune cells under multiple stressors (11, 12). In individuals with stress-induced resilient depression, HDAC3 is extremely upregulated (13). Given that HDAC3 connects stroke and emotion regulation, it is easy to think whether HDAC3 mediates anxiety susceptibility after stroke.

In this study, we first screened molecular changes between nonstroke mice with generalized anxiety disorder (restraint stress, RS) and photothrombotic stroke mice with plus restraint stress (PTS + RS). The RS model simulates the mental conditions of nonstroke populations, while the PTS + RS model is close to stroke patients exposed to adverse situations or stimuli (2, 13). PTS + RS increased the degree of susceptibility to adverse experiences compared with the RS group. In detail, inflammation overactivation, especially microglial activation, is a prominent characteristic of PTS + RS group. Furthermore, we found that the epigenetic remodelling enzyme HDAC3, which mediates the inflammation pathway, was mainly upregulated in microglia of the damaged cortex. HDAC3 inhibition suppresses the inflammatory reaction and regulates the Cox1-EP2 network to influence anxiety susceptibility acquisition. In addition, HDAC3 inhibition enhances mTOR signalling and GSK3 pathway regulation, exerting neuroprotective effects. Finally, we established that HDAC3-mediated p65 deacetylation modifies the activation of downstream NF-κB target genes involved in prostaglandin synthesis. Simultaneously, we developed a device of gamma flicker stimulation for the treatment of PTS + RS, finding that it can relieve anxious behaviour of PTS + RS by modulating the HDAC3/Cox1/EP2 network. Therefore, our findings reveal role of the HDAC3/Cox1/EP2 signalling network in susceptibility to poststroke anxiety and suggest that gamma oscillation can be a useful therapy in alleviating susceptibility to additional stress exposure after stroke.

## Materials and Methods

### Experimental Subjects

Male C57BL/6 mice (18-20 g; Charles River, Beijing, China) were housed under standard conditions (22 ± 2°C, 50–70% humidity) and maintained on a 12-hour light/dark cycle with food and water available. All animal procedures involving this study were approved by the University Committee on Animal Care of Xiamen University and conducted in accordance with the guidelines for Animal Use of the National Institutes of Health.

### Cells

Primary microglia were obtained from the brains of postnatal Days 1~2. Briefly, ice-anaesthetized mice were pre-disinfected and decapitated quickly. After removing the meninges and cerebella in HBSS buffer, the isolated brain tissues were disassociated with a sterile pipette by pipetting up and down several times. Then, homogenates were filtered through a 70-µm cell strainer and centrifuged at 550 g for 5 min. Homogenates were resuspended with 10% heat-inactive foetal bovine serum (FBS, Gibco, 10270-106) DMEM culture medium and transferred into T75 flasks for culturing overnight. Next, DMEM culture medium containing GMSF was added on the second day. Several days later, primary microglia were harvested by shaking flasks at 150 rpm for 15 min. For the murine microglial cell line, BV2 cells were selected for this study.

### Drugs

RGFP109 (RGFP109, Selleck Chemicals) was dissolved in DMSO and diluted in 30% (w/v) hydroxypropyl-β-cyclodextrin (#HK388-5g, Bio Basic Inc., Toronto, Canada) with 100 mM sodium acetate (pH 5.4). Then, RGFP109 was administered subcutaneously once per day for 2 weeks with 60 mg/kg dose.

### Photothrombotic Stroke Mouse Model

After mouse was anaesthetized with isoflurane, the skull was exposed. Then, 100 mg/kg Rose Bengal (Sigma Chemical Co., St. Louis, MO, USA) solution was intraperitoneally injected before illumination, the mouse was fixed in a stereotaxic device, and the range of the skull window was determined (posterior 0.3~2.3 mm and right 0.5~3.0 mm to the bregma). The cold light source (Gohecho Company, Model B5200) was placed as close as possible to the exposed skull and illuminated for 15 min.

### Spatial Restraint Stress Model

Experimental group mice were individually restrained in a well ventilated 50-ml centrifuge tube for 21 days from 9 am to 11 am for 2 h. After restraint stress, experimental mice were removed from the tube and returned to their cages with free access to water and food. Mice in the sham group remained undisturbed in their original cages.

### Cell Inflammatory Stress Model and Intervention Details

Briefly, primary microglial cells or BV2 cells were primed with LPS (500 ng/mL Sigma, L-2880) for 4 h in heat-inactivated 10% foetal bovine serum DMEM, followed by stimulation with 2 mM ATP (Roche, 39251728) for 30 min or not.

### Gamma Visual Stimulation Exposure

Gamma flicker stimulation device was made according to previous reports (14, 15). Before visual stimulation exposure, mice were habituated and transferred from their home cage to a similar cage without bedding in a dark room for 3 h. Then, animals were exposed to LED lights flashing at 40 Hz frequency (12.5 ms light on, 12.5 ms light off) for 2 h. Poststroke anxiety mice were treated with gamma flicker stimulation for at least 21 consecutive days according to protocol 1 or protocol 2 (**Figure S9D**).

### Tissue Preparation, Histology and Imaging

For TTC staining. Mice were anaesthetized with isoflurane and decapitated, and then the brain was obtained. After frozen in -20°C for 20 minutes until the brain became hard, the brain was cut into 1-mm thick slices. Slices were immediately incubated in 1% 2,3,5-triphenyltetrazolium chloride (Sigma–Aldrich Co., St. Louis, MO, USA) at 37°C for 15 min. Then, infarction volume was qualified by ImageJ software.

Mice were anaesthetized and perfused with ice-cold PBS followed by cold 4% paraformaldehyde. Brains were submerged under PFA for 2 h or 12 h. PFA-fixed tissues were transferred into 20% sucrose and 30% sucrose sequentially for 6 h. After OCT embedding, 20-μm sagittal sections of the brain were collected using a cryostat (Leica, CM1950). Thick cryosections were performed with heat-induced antigen retrieval at 95°C (10 mM sodium citrate, 0.05% Tween-20) and then permeabilized at room temperature (RT) with blocking buffer (5% BSA containing 0.2% Triton-X 100 (Sigma, A2153 and T8787)). Primary antibodies were applied at different dilutions in 1% blocking solution overnight at 4°C. After washing with PBS three times, Alexa Fluor-conjugated secondary antibodies were added. Then, slices were mounted with mounting medium containing DAPI (Vector labs H-1000). For confocal microscopy, all images were captured by a Leica TCS SP8 with a Z-stack model. Imaris software (X64 9.2.0) was utilized for microglia 3D reconstruction, surface rendering and filaments with other structural components. Microglia somatic size and process length were analysed with Image J. Colocalization analysis and fluorescence intensity were obtained with Imaris and Leica TCS SP8, respectively.

For cell immunofluorescence, cold PFA-fixed cells (2%) were incubated with blocking buffer for 1 h at room temperature. Primary antibodies were incubated for 6~8 h; secondary antibodies were used later. For confocal microscopy, all images were captured by a Leica TCS SP8.

### Stereotaxic Surgeries and Microinjections

After mice were anaesthetized, the pipette was positioned above the injection site. Injection was performed by lowering the needle rapidly and firmly, after opening or penetration of skull. After penetration, the injection depth was adjusted to the desired location (CeM: AP: -1.4mm; ML: ± 3.5 mm, DV: -5.1 mm), the shRNA or control vehicle was injected unilaterally into the amygdala using a stereotactic injection apparatus (RWD Life Science, Shenzhen, China).

### Separation of Cytoplasmic and Nuclear Proteins

Cells were collected, washed with ice-cold PBS, and lysed with 500 μL buffer A (10 mM HEPES-KOH (pH 7.9), 10 mM KCl, 0.1 mM EDTA, 0.1 mM EGTA, 0.125% NP40, and 1x proteinase inhibitor cocktail) for 20 min at 4°C and agitated it regularly. Then, the contents were centrifuged at 12000 g for 5 min at 4°C. Collected the supernatants (containing the cytoplastic proteins) carefully and washed the precipitate with 1 ml buffer A. Further, the precipitate was lysed by 500 μl RIPA. Finally, the cytoplasmic proteins and nuclear proteins were analysed by western blotting.

### Western Blotting

All proteins samples including cell samples and tissue samples were dissolved into SDS. After tissue being homogenized and lysed, the protein concentration was measured by BCA protein assay kit (Thermo Fisher Scientific) to reach the equal amounts. 8% to 10% SDS-PAGE gel were used when electrophoresis. Then protein strips were transferred onto PVDF membranes (Millipore), followed by incubation with primary antibodies for overnight at 4°C. Using the 1 x TBST to wash out of the excess antibodies, before incubated membranes with HRP-conjugated secondary antibodies for 1 h at room temperature. Detailed antibody information is listed in **Supplementary Table 1**.

### Immunoprecipitation (IP)

For exogenous IP, human embryonic kidney (HEK293T) cells were cultured in DMEM (Life Technologies) containing 10% FBS. The plasmids were transfected with polyethylenimine (PEI, 10 μM). Collected the lysis of the transfected cells in IP buffer (50 mM Tris-HCl pH 7.4, 150 mM NaCl, 1 mM EDTA, 1 M EGTA, 2.5 mM sodium pyrophosphate, 1 mM sodium orthovanadate and 1% Triton X-100 and PMSF (BBI Life Sciences, 329986, 1:100)) on ice. After centrifugation (12000 g, 4°C), 1 μg anti-HA, anti-Flag or anti-Myc was added to the supernatant overnight with gentle rotation. Then 10 μl protein A/G agarose (Yeasen, 36403ES03) resin was added and rotated at 4°C for at least 3 h. After washing with IP buffer, the beads were boiled in 1× SDS sample buffer. Empty vectors or anti-IgG were used as control.

For endogenous immunoprecipitation, tissue was directly lysed in RIPA buffer (50 mM Tris–HCl, 150 mM NaCl, 1 mM EDTA, 1% Triton, 1% Na deoxycholate, 0.1% SDS) supplemented with the cocktail (Roche, 1:1000) and PMSF (1:100) of protease. Cell lysates were incubated with immunoprecipitation antibodies (HDAC3, HDAC1, HDAC2 and p65) at 4°C for 4 hours, followed by overnight incubation with protein G beads. After the beads were washed, the immunoprecipitated protein complex was processed for western blotting with nonspecific IgG as a negative control. Detailed antibody information is listed in **Supplementary Table 1**.

### Quantitative PCR

Tissue or cells were treated with TRIzol reagent (Life Technologies, Carlsbad, CA, USA #15596018) according to the manufacturer’s protocols. A First-Strand cDNA Synthesis Kit (Promega) was used to reverse transcripts. The cDNA was diluted to 10 times with ddH_2_O. Then, cDNA from various groups was quantified by using Fast SYBR Green Master Mix (Thermo Fisher) and a Bio-Rad Real-Time PCR System (Roche, Basel, Switzerland). The relative gene expression level was normalized to that of β-actin. Detailed premiers are listed in **Supplementary Table 1**.

### PGE_2_ Measure

Brain tissue samples were stored at -80°C. The next day, the tissue was homogenized in lysis buffer (0.05 M Tris-HCl, 0.1 M NaCl, and 0.2 mM EDTA, pH 7). The homogenates were centrifuged at 1700 g for 15 min at 4°C. PGE_2_ in the supernatant was measured by a PGE_2_ ELISA kit according to the manufacturer’s protocol (Cayman Chemical, 514531).

### RNA-Seq Analysis

RNA-seq was carried out by the UCLA Neuroscience Genomics Core (UNGC). Samples were pooled and barcoded. The library was prepared using Nugen Ovation RNA Ultra Low Input + Kapa Hyper. Preparation included 120-bp paired-end reads, and the sequencing run was carried out over 5 lanes. On average, 55 million reads were obtained. Reads were aligned to the mouse GRCm38 reference genome using STAR (v.2.4.0), and an average uniquely aligned rate of 80.9 ± 1.1% (mean ± s.e.m.) was obtained. Read counts for RefSeq genes (mm10) were generated by HTSeq v.0.6.1. Low-count genes were filtered, and fragments per kilobase per million mapped reads (FPKM) values were generated. Differentially expressed genes were identified using the Limma package.

### Flow Cytometry

After the mice were anaesthetized, the hippocampus was carefully removed and immediately submerged in serum-free DMEM. The tissue was disassociated with a sterile pipette by pipetting up and down gently several times and incubated at 37°C for 30 min. Then, 550 g was centrifuged for 5 min. Precipitates were filtered through a 70-µm cell strainer. Cells were resuspended in FACS buffer (3% BSA, EDTA), and fluorescent antibodies (CD45-PE-Cy7, CD11b-APC and Ly6G-Fluor^TM-488^) were added for 30 min. After incubation, cells were detected by flow cytometry (Biosciences, Quanteon) and analysed by Flow Jo (Version 10.0).

### Cloning and Plasmid Construction

Mouse *Hdac3*3 (gene ID: 8841), mouse *p65* (gene ID: 19697) and mouse *Kat5* (gene ID: 81601), *Kat8* (gene ID: 67773), *Kat2a* (gene ID: 14534), *COX1* (gene ID: 17708), and *Cbp* (gene ID: 94212) cDNAs were cloned into insect cell expression vectors, pcDNA3.3-Myc, pcDNA3.3-HA and pcDNA3.3-Flag. shEP2 was cloned in pLKO.1, which was modified to add GFP after the inserted fragment (see **Supplementary Table 1**).

### Small Interference RNA

Small interfering RNA targeting *Hdac3* was purchased from BiosMed Technology Co., Ltd. Detailed information is provided in the Supplementary Information. 40 nM oligonucleotides were transfected into mouse primary microglia with 5.5 μl Lipofectamine RNAi MAX reagent (13778, Invitrogen). The mixture was incubated at RT for 5 minutes. The siRNA-RNAi MAX complex was added to 1ml 5% FBS DMEM cultures in a cell incubator for 48 hours at 37°C.

### Lentivirus Production and Transduction

Short-hairpin RNA (shRNA) was constructed with recombinant pLKO.1 as mentioned above, and plasmids were co-transfected into HEK293T cells with the packaging plasmid pSPAX2 and enveloping plasmid pMD.2G using the PEI-based transfection method to produce lentiviral virus for 8 h. Then, serum-free medium was replaced with serum-containing DMEM, and the supernatant was harvested after 72 h and 36 h. Virus was collected through 70000 g centrifugation at 4°C and titrated by infecting HEK293T cells.

### Behavioural Testing

#### Open Field Test (OFT)

To measure the anxious behaviour and general activity of animals, we characterized mouse behaviour as they freely explored an open-field plastic chamber (50-cm width × 50-cm length × 50-cm height). Around three months old mice were placed in this area for 10 min. Total distance and time spent in the central region was recorded (Video camera system, smart 3.0).

#### Elevated Plus Maze Test (EPM)

Mice were placed into the centre quadrant of a 4-arm maze with two open arms without walls and two closed arms with walls. The mice were placed in the centre and faced a closed arm at the start of a trial. This software (Smart 3.0) tracked the amount of time the mice spent in the closed arms versus the open arms throughout a 5-min session.

#### Tail Suspension Test (TST)

Mice were suspended by their tails from an acrylic bar (15 cm in diameter, 30 cm in height) for five minutes. Escape-related behaviour was assessed, where immobility duration (%) during the 5 min suspension period was recorded (Smart 3.0).

### Electrophysiology

The mice brains were rapidly removed, placed in ice-cold artificial hypertonic glucose solution (120 mM sucrose, 2.5 mM KCl, 10 mM MgSO_4_, 1.25 mM NaH_2_PO_4_, 26 mM NaHCO_3_, 10 mM D-glucose, 64 mM NaCl, and 0.5 mM CaCl_2_) after mice were anaesthetized with isoflurane rapidly. Meanwhile, artificial hypertonic glucose solution was bubbled with carbogen (95% O_2_ + 5% CO_2_). Brain slices were cut (400 µm thick) by Leica VT1200S vibratome. The slices were incubated in ACSF (3.5 mM KCl, 120 mM NaCl, 1.3 mM MgSO_4_, 10 mM D-glucose, 1.25 mM NaH_2_PO_4_, 26 mM NaHCO_3_, and 2.5 mM CaCl_2_), and bubbled with carbogen at 32°C for 1 hour and recovered at RT for at least 1 hour with consistent carbogen supply. The Schaffer collateral inputs to the CA1 region pathway were selected for LTP recording. Baseline responses were acquired every 20 s with a stimulation intensity that yielded 30% of the maximum response. After a 20-min stable baseline recording, LTP was induced by high-frequency stimulation (two trains of 100-Hz stimuli with an interval of 30 s), followed by continued recording for 60 min.

### Quantification and Statistical Analyses

All outcomes presented in this manuscript are displayed as the mean ± SEM or SD. Statistical analysis was carried out with a two-tailed unpaired method (Student’s *t*-test) when we were faced with two independent groups, and one-way ANOVA for multiple groups with single variance, all calculated with GraphPad Prism 8. Significant statistical figure outcomes must obey a p value upper bound less than 0.05. In addition, the statistical legends marked in the chart are explained as follows (* indicates p < 0.05; ** indicates p < 0.01; *** represents p < 0.005).

## Results

### Additional Stress Exposure After Cortex Infarction Increases the Degree of Susceptibility to Adverse Experiences

**Figure 1:**
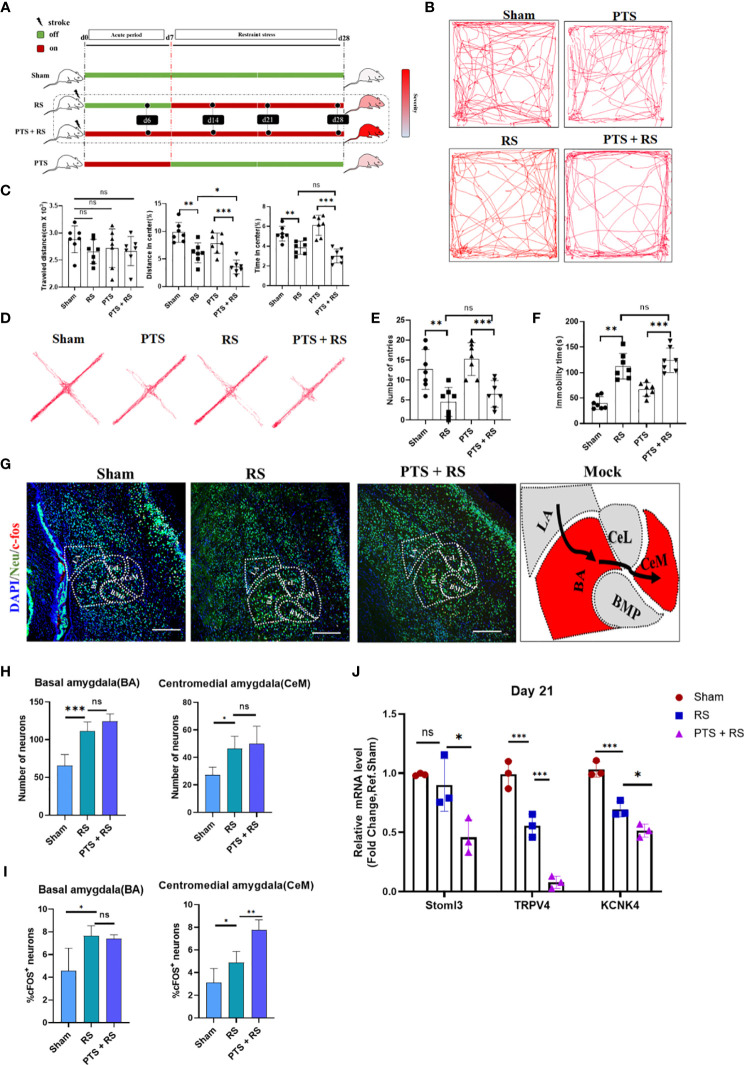
Additional stress exposure after cortex infarction increases the degree of susceptibility to adverse experiences. **(A)** Systematic experimental design. The time points of the various groups are shown above, and the days mentioned in this article all obey this principle. **(B)** Representative tracks of the open-field test (OFT) between sham, photothrombotic stroke (PTS), restraint stress (RS) and photothrombotic stroke plus restraint stress (PTS + RS) mice on day 31 after induction of the model. **(C)** Statistical analysis of travel distance, percentage of distance travelled in the centre and time spent in the centre area (N = 7 per group). **(D)** Elevated plus-maze (EPM) represents four groups of motivation tracks. **(E)** Number into open arm was described on Day 33 after OFT finish (N = 7 per group). **(F)** Immobility time spent was tested by the tail suspension test on Day 34 after EPS was finished. **(G, H)** Amygdaloid nucleus staining of neurons (Neu) and c-fos **(G)** (scale bar = 200 µm); statistical data **(H, I)** were based on the number of neurons and percentage of c-fos-positive neurons in the corresponding nuclear groups (basal amygdala (BA) and centromedial amygdala (CeM)). **(J)** qRT-PCR outcomes of amygdaloid between the sham, PTS and PTS + RS groups on Day 21 (acute period of model), and the Q-PCR assay was performed at least three times. The animal number of behavioural tests in **(A–F)** was 7 per group. The qRT-PCR assay was performed in at least three independent experiments. The data mentioned above are presented as the means ± SEM and were analysed by one-way ANOVA with Bonferroni *post hoc* test. (ns, no significance; *p < 0.05; **p < 0.01; ***p < 0.005).

We first characterized the difference in negative emotion acquisition between sham, restraint stress (RS), photothrombotic stroke and photothrombotic stroke plus restraint stress (PTS + RS) mice on Day 31 after induction of the model. To approximately correct the infarction level baseline between PTS and PTS + RS and avoid group error induced by lesion variation, the infarct volume between PTS and PTS + RS was not significantly different on the second day of the acute phase after modelling (**Figures S1A, B**). From the day of cortex infarction, behaviour test was performed at Day 31 from the initiation of the inducing model (**Figure 1A**). According to the outcomes of the open-field test (**Figures 1B, C**), in contrast to sham mice, RS and PTS + RS mice in both groups showed reduced interest in exploring the central region, featuring a lower percentage of distance and time spent in the centre. The elevated plus maze (EPM) test and tail suspension test showed that PTS and PTS + RS mice were more susceptible to adverse emotions (**Figures 1D–F**). The symptoms of negative emotions, including depression and anxiety, were closely correlated with stress intensities. PTS + RS mice had more severe emotional dysfunction than PTS mice. In addition, PTS + RS mice had a greater anxiety inclination than the RS alone group. Next, we tested anxious behaviour during the acute period of PTS on the fourth day. We discovered that PTS solely contributes to the sensitivity of anxious behaviour despite the amygdala not being directly damaged by ischaemic damage (**Figures S1C, D**). Therefore, we speculate that cortex infarction may increase some endogenous molecules that aggravate adverse experiences and that additional stress amplifies this biological behaviour according to several outcomes, including reflecting anxious behaviour test tracks, damage-associated cortex situations and severe stress-induced intestinal disorders (**Figures S2A–G**).

To understand the mechanism underlying this abnormal behaviour extraverted by cortex infarction itself, we sought to identify the relevant neuronal circuits regulating adverse experiences. The immediate early gene c-Fos is a candidate marker reflecting neuronal activity. The formation of anxiety information is vividly related to the amygdala. According to previous reports, BA (basal amygdala) but not LA (lateral amygdala) neurons project to the centrolateral (CeL) and CeM nucleus. The CeM is regarded as the primary output nucleus of the amygdala, in which BA→CeM directly mediates downstream targets about anxiety-related behaviours (16). RS induced a classical anxiety model, and we observed that compared with the sham group, RS mice exhibited neuronal overactivation of BA and CeM, same as PTS + RS mice. But there is no number difference of neurons in BA or CeM between RS and PTS + RS (**Figures 1G, H** and **Figures S3A, B**). However, this phenomenon cannot explain why the PTS + RS group acquires more severe adverse emotions than the RS group. Therefore, c-Fos ^+^ Neu (Neuron)^+^ percentage was performed to analysis deeply. Results showed that CeM c-Fos ^+^ Neu^+^ percentage in the PTS + RS group was higher than the c-Fos ^+^ Neu^+^ percentage in the RS group, while no difference in BA (**Figure 1I** and **Figures S3A, C, D**). Potassium channels participate in resting neuron overactivation and are reported to be dysfunctional in adverse depression experiences and anxious behaviour (17, 18). The amygdala of PTS + RS displayed lower potassium channel like Stoml3, TRPV4 and KCNK4 expression according to qPCR results, compared with the RS mice (**Figure 1J**) (18). So, amygdala as output nucleus of anxiety behaviour is influenced molecularly by distant infarcted cortex because of no direct ischemic injury. And the difference of anxiety susceptibility between poststroke anxiety and generalize anxiety disorder was well simulated respectively by the PTS + RS mice and RS mice. These results further remind us that when individuals were exposed to the additional same intensity of environmental stress, cortex infarction injury increases the degree of susceptibility to adverse experiences.

### Inflammation Overactivation Is a Prominent Characteristic of Poststroke Anxiety

**Figure 2:**
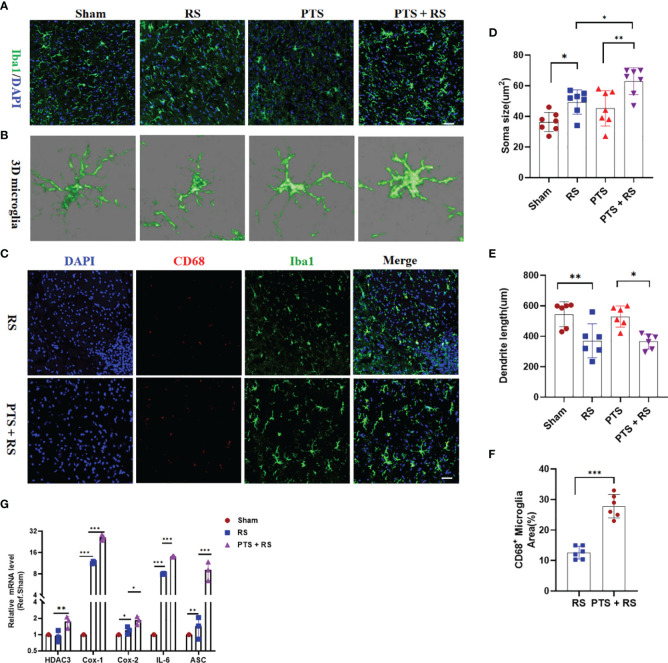
Inflammation overactivation is a prominent characteristic of poststroke stress. **(A)** Histological analysis of cortical microglia from sham, PTS, RS and PTS + RS mice was performed by staining for Iba1 (green) and GFAP (yellow) on Day 28. (Scale bar = 50 µm). **(B)** 3D microglia reconstruction was performed by Imaris. **(C)** Histological analysis of activated microglia from RS and PTS + RS mice was performed by staining for CD68 (red) and GFAP (Iba1). (Scale bar = 50 µm). **(D, E)** Statistical analysis of microglial morphology (soma size and dendrite length) from four groups. **(F)** Statistical analysis of the percentage of CD68-positive microglia from RS and PTS + RS mice. **(G)** qRT-PCR outcomes of relative inflammatory cytokines from sham, RS and PTS + RS mice on the day of 21. The data mentioned above are presented as the means ± SEM and were analysed by one-way ANOVA with Bonferroni *post hoc* test or Student’s t-test when comparing 2 groups. *p < 0.05; **p < 0.01; ***p < 0.005).

Inflammatory signalling within the central nervous system (CNS) contributes to motivational and affective states during many pathological conditions from humans to rodents (19, 20). Imaging studies and post-mortem analysis of depression or long-term adverse patients indicate that microglial overactivation is prominent (14, 15). Microglia have been reported to mediate inflammatory reactions to regulate mood (21). To illuminate the inner cell biological mechanism between the PTS + RS mice and RS mice, microglial activation was included for analysis. We found that microglia in the damaged cortex were overactivated in the PTS + RS group, with features of larger cell soma size and shorter process length according to microglial 3D rebuilding (**Figures 2A–E**). Inflammation, phagocytosis and oxidative stress are three main characteristics of microglia, participating in the stress-susceptible behavioural phenotype (22). Further, we founded that percentage of CD68-positive microglia per area was higher in the PTS + RS group than the RS group (**Figures 2C, F**). And inflammatory cytokines such as Cox-1, Cox-2, ASC and IL-6 were increased more in the PTS + RS group (**Figure 2G**). These results indicate that inflammation overactivation, especially microglia-mediated activation, is a prominent characteristic of poststroke anxiety, compared with generalize anxiety disorder. So, phobic anxiety susceptibility after stroke is closely connected with activated microglia.

### Differential Expression Profiles of Transcripts Reveal the Details of Molecular Adaptation in the Brain After Stroke Accompanied by Additional Stress

**Figure 3:**
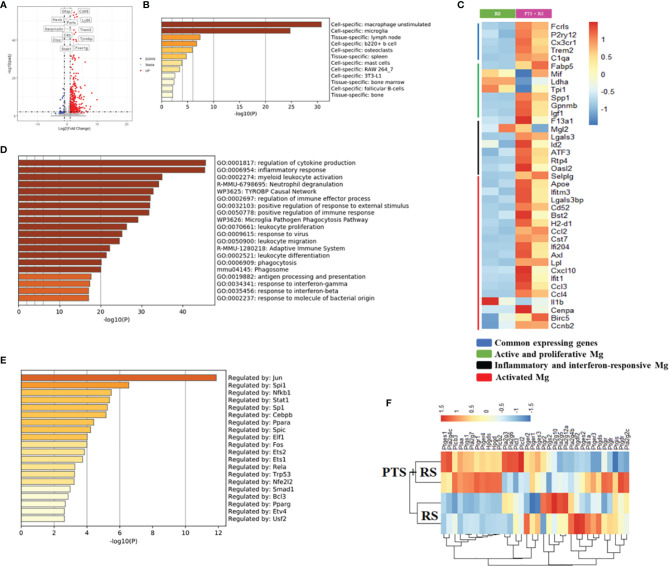
Differential expression profiles of transcripts reveal the details of molecular adaptation in the brain after stroke accompanied by additional stress. **(A)** Volcano plot of nearby damaged cortex transcriptome differential genes of RS and PTS + RS. **(B)** Bar graph of enriched cell types of upregulated genes of the PTS + RS group compared with the RS group, coloured by p value. **(C)** Heatmap of various microglia unique marker genes between RS and PTS + RS. **(D)** Pathway and process enrichment analysis of upregulated genes in the PTS + RS group compared with the RS group. **(E)** Bar graph of mainly enriched transcription factors of the PTS + RS group compared with the RS group, coloured by p value. **(F)** Gene expression changes associated with prostaglandin synthesis pathways.

To illustrate inner molecular changes between two groups, damaged cortex tissue was extracted for RNA-seq analysis. The results showed 396 upregulated genes and 32 downregulated genes (**Figure 3A**). Upregulated genes are usually regarded as adapted changes under external stress. A summary of the enrichment analysis of upregulated genes in PaGenBase indicated that unstimulated macrophages and microglia were greatly enriched in the brain (**Figure 3B**) (23), consistent with microglia analysis in **Figure 2**. Microglia are prominent immune cells of the central nervous system and are closely connected with homeostasis and cognition and neurogenesis and regulate synaptic development and plasticity (24). To further explain the reason for the molecular changes, microglia maker genes were included for analysis. Common microglial genes, active and proliferative microglia, inflammatory and interferon responsive microglia and activated microglia were greatly upregulated in PTS + RS to some extent (**Figure 3C**). Stroke plus restraint stress also leads to other pathways: 1) Oxidative stress and redox pathways are out-of-balance; 2). Production of nitric oxide is disordered; 3). Mitochondrial ATP synthesis is coupled with electron transport pathway dysfunction (**Figures S4A–C**). These upregulated genes analysed integrally were greatly enriched in the regulation of cytokine production, inflammatory response and leukocyte infiltration (**Figure 3D** and **Figure S4D**). Besides, Kyoto Encyclopedia of Genes and Genomes (KEGG) analysis indicated that pathways about immune interactions, antigen presentation and inflammatory responses (NOD-like receptor signalling pathway, antigen processing and presentation) were enriched highly in PTS + RS group (**Figure S4E**). These results prove again that microglia promote the progression of PTS + RS when compared with the RS group alone. Stroke may trigger microglia overactivation, promoting the susceptibility of individuals to environmental stress. Sustained response of pathway changes can be attributed to the enrichment of epigenetic transcription factors (**Figure 3E**). The most significantly enriched genes were Jun, Spi1, Nfkb1, Stat1, etc. The NF-κB pathway intercrosses many paths mentioned above and has been reported to indirectly lead to the symptoms of anxiety-like behaviour. In line with these findings, the NF-κB pathway-intercrossed pathway, such as the prostaglandin-mediated synthesis pathway, changed greatly (**Figure 3F**). Prostaglandin from activated microglia elicits a negative affective state (20). In summary, we hypothesized that cortical infarction is a trigger for microglia to produce prostaglandin, eliciting susceptibility to additional stress exposure.

### HDAC3 Is a Candidate Molecule Participating in Disease Progression and Vividly Related to Prostaglandin Production in the Brain Exposed to Stress

**Figure 4:**
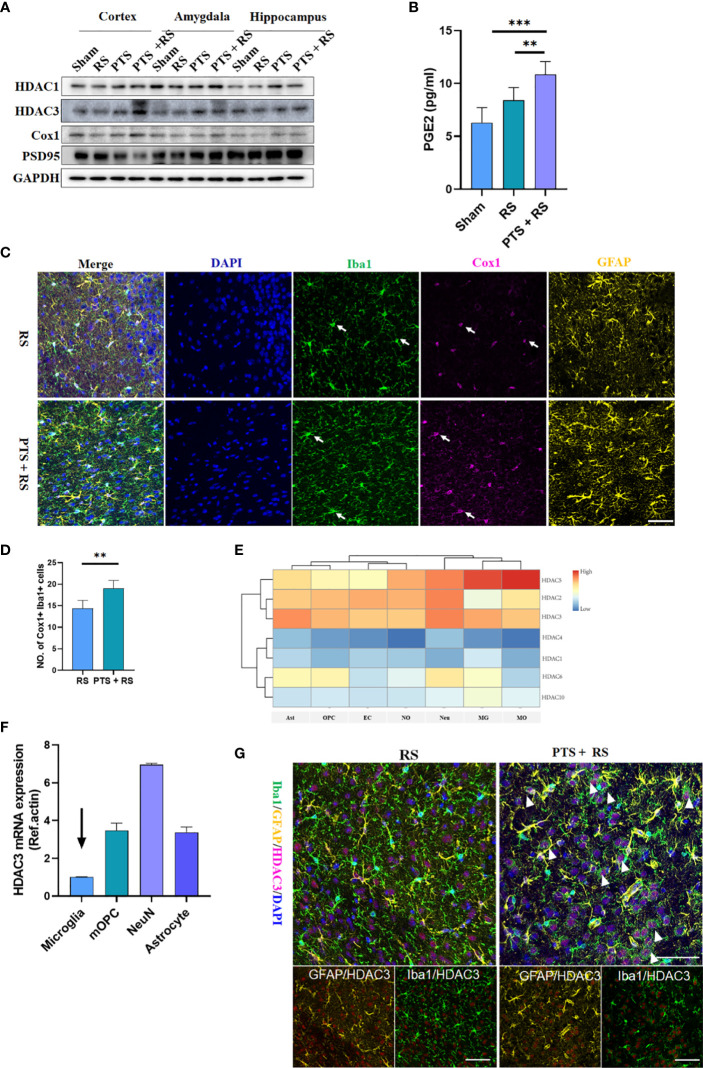
HDAC3 is a candidate molecule participating in disease progression and vividly related to prostaglandin production in the brain exposed to stress. **(A)** Representative Western blot of HDAC3, HDAC1, Cox1 and PSD95 in the cortex, hippocampus and amygdala after 14 d in various groups. **(B)** Prostaglandin levels in the four groups on Day 21. **(C)** Immunofluorescence of Cox1, Iba1 and GFAP in the RS and PTS + RS groups. (Scale bar = 50 µm). **(D)** Statistical analysis of the number of Cox1 ^+^ Iba1 ^+^ prostaglandin-accumulating microglia in various cortical sites. **(E)** Relative expression levels of histone deacetylase (HDAC1, HDAC2, HDAC3, HDAC4, HDAC5, HDAC6 and HDAC10) in astrocytes (Ast), oligodendrocytes (OPC), endothelial cells (EC), newborn oligodendrocytes (NO), neurons (Neu), microglia (MG) and mature oligodendrocytes (MO) according to a single-cell comparison database. **(F)** qRT-PCR outcomes of HDAC3 relative expression levels in microglia, mature oligodendrocytes, neurons and astrocytes. **(G)** Immunofluorescence of HDAC3, Iba1 and GFAP in the RS and PTS + RS groups. (Scale bar = 50 µm). Arrows represent randomly selected co-located reference points. The data mentioned above are presented as the means ± SEM and were analysed by one-way ANOVA with Bonferroni *post hoc* test or Student’s t-test when comparing 2 groups. **p < 0.01; ***p < 0.005).

NF-κB pathway can be regulated closely by histone deacetylases (HDACs). HDACs have been reported to be involved in regulating the inflammatory pathway, cell survival, memory and emotion regulation (10, 12, 25, 26). However, the relationship between HDACs and prostaglandin remains unclear. We obtained cortical (infarct side), hippocampal and amygdala tissues from the sham, RS, PTS and PTS + RS groups for protein detection. We found that HDAC3 in damaged cortex of PTS or PTS + RS group was moderately higher than sham and RS groups. While, the difference of HDAC1 in these groups is weak. We further detected the prostaglandin synthesizing enzyme cyclooxygenase-1 (COX1; encoded by the gene Ptgs1), the rate-limiting enzyme for the conversion of arachidonic acid into prostaglandin. The expression of Cox1 was most significant in the injured cortex, while Cox1 in the hippocampus and amygdala were not significantly expressed. Simultaneously, we observed that the expression level of PSD95 in the damaged cortex changed prominently (**Figure 4A**). These results indicate that stroke in the cortex is responsible for epigenetic inductions, such as HDAC3 and Cox1. Furthermore, damaged cortex homogenates were harvested for PGE_2_ detection because PGE_2_ is a major metabolite involved in the regulation of negative emotion (20). We found that RS moderately increased local PGE_2_ production, while PTS + RS dramatically boosted PGE_2_ production during the subacute period (**Figure 4B**). Moreover, the PTS + RS group exhibited prostaglandin-enriched microglia but not astrocytes (**Figures 4C, D**) (27). These similar results were in concert with our transcriptome bioinformatics results (**Figure 2**). Next, we founded that HDACs were widely expressed in various cells in the CNS, as shown by our database comparison results (**Figure 4E**) (27, 28). qPCR outcomes of various primary cells, including microglia, mOPCs, neuron and astrocytes, indicated that the basic expression level of HDAC3 was low (**Figure 4F**). However, our protein expression level of the four groups showed that HDAC3 was visibly upregulated. Therefore, we believe that the expression of HDAC3 varies in different cells. Based our previous evidence of inflammation-related pathways, inflammatory cells such as astrocytes and microglia were included for analysis of HDAC3 expression. Surprisingly, we found that PTS + RS augmented HDAC3 expression in microglia compared with RS instead of astrocytes (**Figure 4G**).

### Inhibiting HDAC3 Alleviates Inflammation-Induced Aversion and Exerts Neuroprotective Effects by Modulating Microglial Activation and Prostaglandin Production

**Figure 5:**
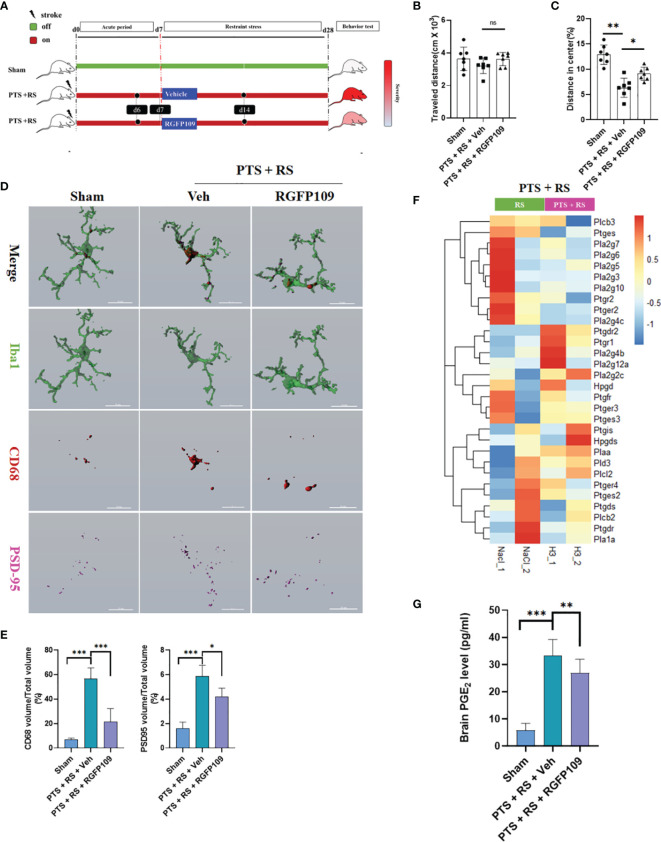
Inhibiting HDAC3 alleviates inflammation-induced aversion and exerts neuroprotective effects by modulating microglial activation and prostaglandin production. **(A)** Systematic diagram of experimental design. Sham mice were used as a control; one group was given the HDAC3 inhibitor (RGFP109), and the other group was given the vehicle in PTS + RS mice for 21 consecutive days from Day 7 to Day 28. **(B, C)** Representative racks of open-field test (OFT) between sham, photothrombotic stroke plus restraint stress (PTS + RS) mice with or without RGFP109 on Day 31 after intervention finishing and statistical analysis (N = 7 per group). **(D, E)** Imaris analysis of PSD95, CD68 and Iba1 in sham, photothrombotic stroke plus restraint stress (PTS + RS) mice with or without RGFP109 on Day 14 **(D)**; **(E)** represents the volume ratio of CD68 or PSD95 in microglia. (Scale bar = 15 µm). **(F)** shows the transcriptome differences in prostaglandin synthesis and metabolic pathways between the two groups. **(G)** Relative prostaglandin content in the damaged cortex from three various groups. The data mentioned above are presented as the mean ± SEM and were analysed by one-way ANOVA with Bonferroni *post hoc* test (ns, no significance; *p < 0.05; **p < 0.01; ***p < 0.005).

To explain the direct effect of HDAC3 on emotion regulation of PTS + RS, the HDAC3 inhibitor RGFP109 was utilized for two weeks when mice were under restraint stress (**Figure 5A**). An open-field test was performed to assess the severity of anxiety behaviour between the PTS + RS (Vehicle) group and the PTS + RS (RGFP109) group. The total travelled distance showed no significant differences. The PTS + RS (RGFP109) group manifested more mild anxiety behaviour with a higher percentage of distance in the centre (**Figures 5B, C** and **Figures S6A, B**). To further determine the biofunction of HDAC3 inhibition on microglia, 3D-rebuilt microglia were analysed to assess the activation of microglia (**Figure 5D** and **Figure S6C**). The percentage of CD68 volume/total microglia volume referred to microglial activation. The results show that RGFP109 supply prominently decreases the percentage of CD68 volume/total microglia volume, compared with PTS + RS (Vehicle) group. Activated microglia usually feature nonselective pruning of synapses, exacerbating further damage to the neural circuit. Engulfed synapse maker protein PSD-95 was quantified. We find that RGFP109 treatment decreases percentage of PSD95 volume/total microglia volume in PTS + RS group (**Figures 5D, E**). Notably, RGFP109 also decreases synapse damage maker C1q expression (**Figures S6C, D**). And RGFP109 improves synapse homeostasis by upregulating p-GSK-3β and GSK-3α/3β but has no contribution to the increase in PSD-95 itself (**Figure S6E**). In addition to the neuroprotective effect of HDAC3 inhibition, prostaglandin synthesis-related genes were widely downregulated (**Figure 5F**). Additionally, the content of PGE_2_ was suppressed with HDAC3 inhibition (**Figure 5G**). Collectively, these data show that inhibiting HDAC3 alleviates inflammation-induced aversion and exerts neuroprotective effects by modulating microglial activation and prostaglandin production.

### HDAC3 Inhibition Suppresses the Inflammatory Reaction and Regulates the Cox1-EP_2_ Network to Influence Anxiety Susceptibility Acquisition

**Figure 6:**
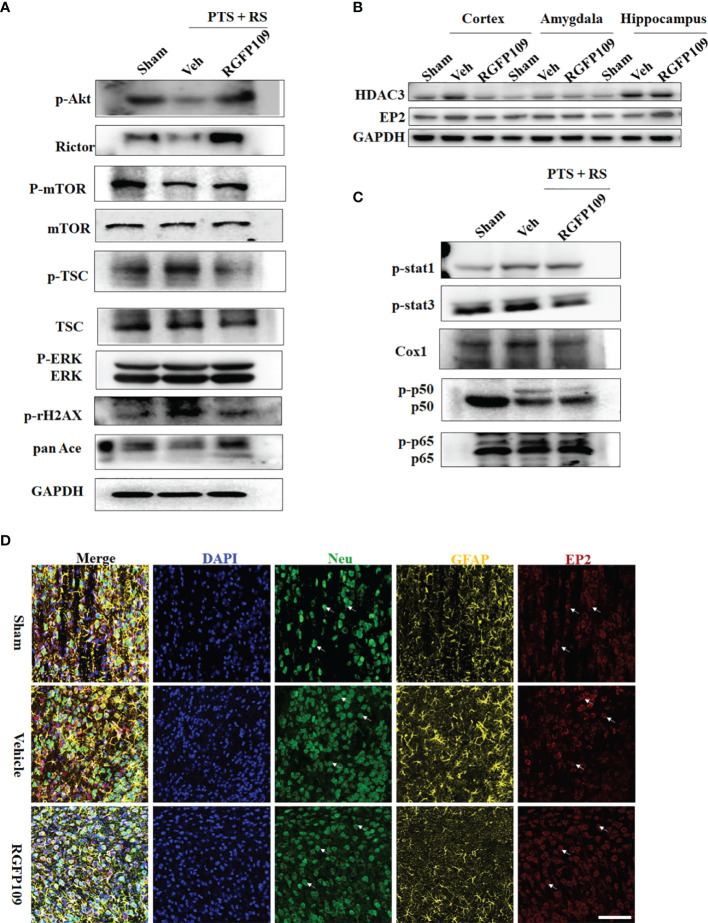
HDAC3 suppresses the inflammatory reaction and regulates the Cox1-EP2 network to influence stress susceptibility acquisition. **(A)** Western blot outcomes of p-Akt, Rictor, p-mTOR, mTOR, p-TSC, TSC, p-γH2AX, pan-Ace, ERK and p-ERK in the sham, PTS and PTS + RS groups. **(B)** Western blot outcomes of HDAC3 and EP2 in the hippocampus, amygdala and cortex in sham rats and PTS + RS rats with or without RGFP109. **(C)** Western blot outcomes of p-Stat1, p-Stat3, Cox1, p-p50, p50, p65 and p-p65 in the sham, PTS and PTS + RS groups. **(D)** EP2 relative expression levels in neurons (Neu) and astrocytes (GFAP) of the sham, PTS + RS with or without RGFP109 groups. Arrows represent randomly selected co-located reference points.

First, to probe the molecular change of HDAC3 inhibition in PTS + RS on synaptic plasticity, Akt-mTOR signalling is described in detail in **Figure 6A**. HDAC3 inhibition with RGFP109 enhanced mTOR signalling by upregulating p-Akt, p-mTOR, and Rictor and downregulating p-TSC expression. Akt-mTOR signalling is a pathway phenotype involved in the function of synapses that activates protein synthesis, alleviating depression or anxiety. Therefore, a causal link between HDAC3 expression disorder and anxiety-like symptoms has been established. However, the pathway phenotype cannot explain how microglial activation impacts neuronal activation, participating in adverse emotion progression. Prostaglandin overgeneration in PTS + RS, compared with its PTS counterpart alone, indicated that HDAC3 inhibition correlated closely with prostaglandin (**Figure 5G**). Previous researchers have found that EP1 and EP2 are two main receptors expressed in neurons (20, 29). Prostaglandin from activated microglia reduces the excitability of striatal neurons, leading to a negative affective state *via* EP1- and EP2-mediated signalling. However, we do not know whether the anxiety susceptibility to stroke plus environmental stress is attributed to the prostaglandin-EP network. To answer this question, first, the experiments described in Sup 5 identified the dynamics of damage-associated cytokines. We found that IL-6, ASC, Cox1 and Cox2 were at a constant high level, with positive reference of IL-6 and ASC proved to influence adverse behaviour (**Figures S5B–E, G**). Naturally, the path of prostaglandin generation is emphasized with increasing attention according to previous data. Furthermore, we detected the relative mRNA levels of prostaglandin receptors (EP1, EP2, EP3 and EP4) in the damaged cortex and amygdala. EP2 was the only receptor upregulated in the acute and subacute stages (**Figure S5F**). EP2 was downregulated by RFP109, accompanied by a decrease in HDAC3 in the amygdala (**Figure 6B**). Next, we detected the location of the EP2 expression of microglia, astrocytes and neurons in the amygdala. The data showed that EP2 was reduced mainly in neurons but not microglia and astrocytes (**Figure 6D**). Then, we utilized lentivirus containing the shEP2 vector to implement stereotactic bilateral amygdala injection. After 2 weeks, the PTS + RS model was conducted. Behavioural tests suggested that EP2 knockdown in the amygdala relieved anxious behaviour acquisition (**Figures S7B, C**). Therefore, we concluded that HDAC3 inhibition attenuates anxiety behaviour by restraining prostaglandin overproduction and downregulating neuronal EP2 expression in the amygdala. However, we did not trace the upstream signalling pathways of HDAC3 in prostaglandin production. We observed that interferon response genes (Stat3, pStat3; Stat1, pStat1) were notably abrogated. In addition, HDAC3 inhibition led to reducing expression of the NF-κB signalling main genes p65, p-p65, p50 and p-p50, along with repressed expression of Cox1 (**Figure 6C**). In addition, synthesizing previous data (**Figure 3**) bolstered the idea that prostaglandin synthesis and inflammatory signalling activation were connected with NF-κB signalling suppression by HDAC3 inhibition.

### HDAC3-Mediated p65 Deacetylation Modifies the Activation of Downstream NF-κB Target Genes *In Vitro*

To clarify whether NF-κB signalling is related to microglia and whether HDAC3 inhibition of prostaglandin production is linked to microglial activation, the amygdala and damaged cortex were included for detailed analysis between microglia and HDAC3 inhibition. A heatmap differential gene expression matrix showed that inflammatory and interferon-responsive microglia were repressed in both the amygdala and cortex after RGFP109 treatment. Activated microglia in the amygdala were also suppressed. Microglia in the infracted cortex represented an active state, such as axon tract-associated microglia and active and proliferative microglia, which may be connected with reconstruction, such as axon guidance, synapse protection and compound synthesis, that forms myelin after ischaemic injury during the subacute stage of PTS + RS (**Figures S8A–C**). According to flow cytometric analysis of neutrophils (CD11b^+^Ly6G^+^) and microglia (CD45^low^ CD11b^high^), HDAC3 inhibition reshaped microglial function by limiting overactivation in the chronic period but not neutrophils (**Figures S9A–D**). Simultaneously, we detected inflammatory cytokines in damaged lesions and reflected microglial subtype genes (**Figures S9E, F**). We found that HDAC3 inhibition reliably alleviated anxiety susceptibility to PTS + RS by remodelling microglial activation. In provisional summary, cortex infarction is a trigger for microglia enriching prostaglandin, eliciting susceptibility to additional stress exposure. HADC3 inhibition reverses microglial overactivation and controls prostaglandin levels, alleviating anxiety susceptibility.

**Figure 7:**
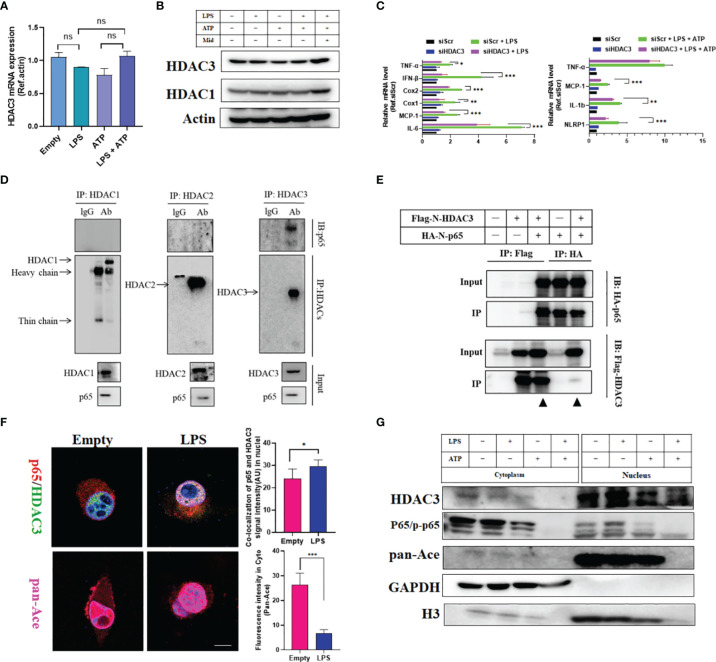
HDAC3-mediated p65 deacetylation modifies the activation of downstream NF-κB target genes *in vitro*. **(A)** qRT-PCR outcomes of primary microglia stimulated with LPS, ATP and LPS + ATP. **(B)** Western blot outcomes of primary microglia stimulated with LPS, ATP, LPS + ATP and LPS + ATP + Midi. **(C)** qRT-PCR outcomes of primary microglia stimulated with LPS and LPS + ATP with or without HDAC3 knockdown. **(D)** Co-IP outcomes of HDACs (HDAC1, HDAC2 and HDAC3) with p65 in primary microglia. **(E)** Exogenous Co-IP outcomes of Flag-N-HDAC3 and Flag-N-p65. **(F)** Cell immunofluorescence colocalization analysis of p65 and HDAC3 with or without LPS stimulation. (Scale bar = 5um). **(G)** Cell nucleus and cytoplasmic fraction separation for detecting the spatial localization of HDAC3 and p65 after various cell stresses. The data mentioned above are presented as the means ± SEM and were analysed by one-way ANOVA with Bonferroni *post hoc* test or Student’s t-test when comparing 2 groups. (ns, no significance; *p < 0.05; **p < 0.01; ***p < 0.005).

Therefore, we hypothesized that HDAC3 directly regulates prostaglandin production in microglia. First, we used an LPS-induced inflammatory model and LPS + ATP to mimic an inflammasome activation model. LPS- or LPS + ATP-stimulated primary microglia did not induce HDAC3 expression at the transcriptional or translational level (**Figures 7A, B**). However, Mdivi-1 (5 µM), a small-molecule inhibitor of mitochondrial fission, moderately increased HDAC3 expression under LPS + ATP challenge. These results were a reminder that HDAC3 is independent of external inflammatory stress but connected with intracellular mitochondrial function. Unfortunately, we did not figure out upstream signalling of HDAC3. To solve downstream pathways linked to inflammatory gene expression, HDAC3 knockdown under LPS stimulation or LPS + ATP stimulation repressed the expression of NF-κB target inflammation-related genes, such as TNF-α, IFN-β, Cox1, Cox2, MCP-1, IL-6, NLRP1 and IL-1β (**Figure 7C** and **Figures S10A, B**). Thus, HDAC3 inhibition blocked NF-κB downstream target genes, including enzymes for prostaglandin synthesis, such as Cox1 and Cox2.

Furthermore, we found higher binding between HDAC3 and p65 (an NF-κB/Rel family transcription factor) in primary microglia than in other HDACs (HDAC1 and HDAC2) (**Figure 7D**) (30). These results were confirmed by exogenous immunoprecipitation and cell immunofluorescence (**Figures 7E, F**). Lysine acetylation at sites 122 and 123 of p65 has been reported to result in the cytoplasmic (Cyto) translocation of p65 from the nucleus (10). As shown in **Figure 7F**, we observed that colocalization between p65 and HDAC3 increased in the nucleus under LPS challenge, accompanied by pan-Ace levels in the cytoplasm. The cytoplasm and nucleus of HDAC3, p65/p-p65, and pan-Ace were analysed by Western blot. Similarly, p65 and HDAC3 both translocated from the cytoplasm to the nucleus under inflammatory stress, and pan-Ace decreased under stress changes in the cytoplasm but not the nucleus (**Figure 7G**). To ensure HDAC3-mediated deacetylation modification of p65, we cloned four acetyltransferases (CBP (CREB-binding protein), Kat8, Kat2a and Kat5) to illustrate which acetyltransferase is the main factor for acetylation of p65. We found that Kat8 was the most highly binding enzyme (**Figure S10C**). Then, we overexpressed HA-p65 and myc-CBP in HEK293T cells with or without Flag-HDAC3. We found that CBP acetylated p65 with high pan-Ace levels, and this acetylation modification could be neutralized by co-expressing Flag-HDAC3 (**Figure S10D**). These results suggest that HDAC3 mediates acetylation of p65, increases its nuclear translocation, and promotes the expression of NF-κB target inflammation-related genes related to prostaglandin synthesis. Therefore, these results can explain why prostaglandin-enriched microglia with high Cox1 expression in PTS + RS are caused by HDAC3/NF-κB/Cox1 signalling activation. With further explanation, prostaglandin, especially PGE_2_ overproduction from activated microglia, acts on neurons in the amygdala *via* EP2, increasing anxiety susceptibility acquisition in the PTS + RS group compared with the RS group.

### Gamma Visual Stimulation Alleviates Susceptibility to Additional Stress Exposure After Cortex Infarction by Modulating HDAC3/Cox1/EP2 Expression

**Figure 8:**
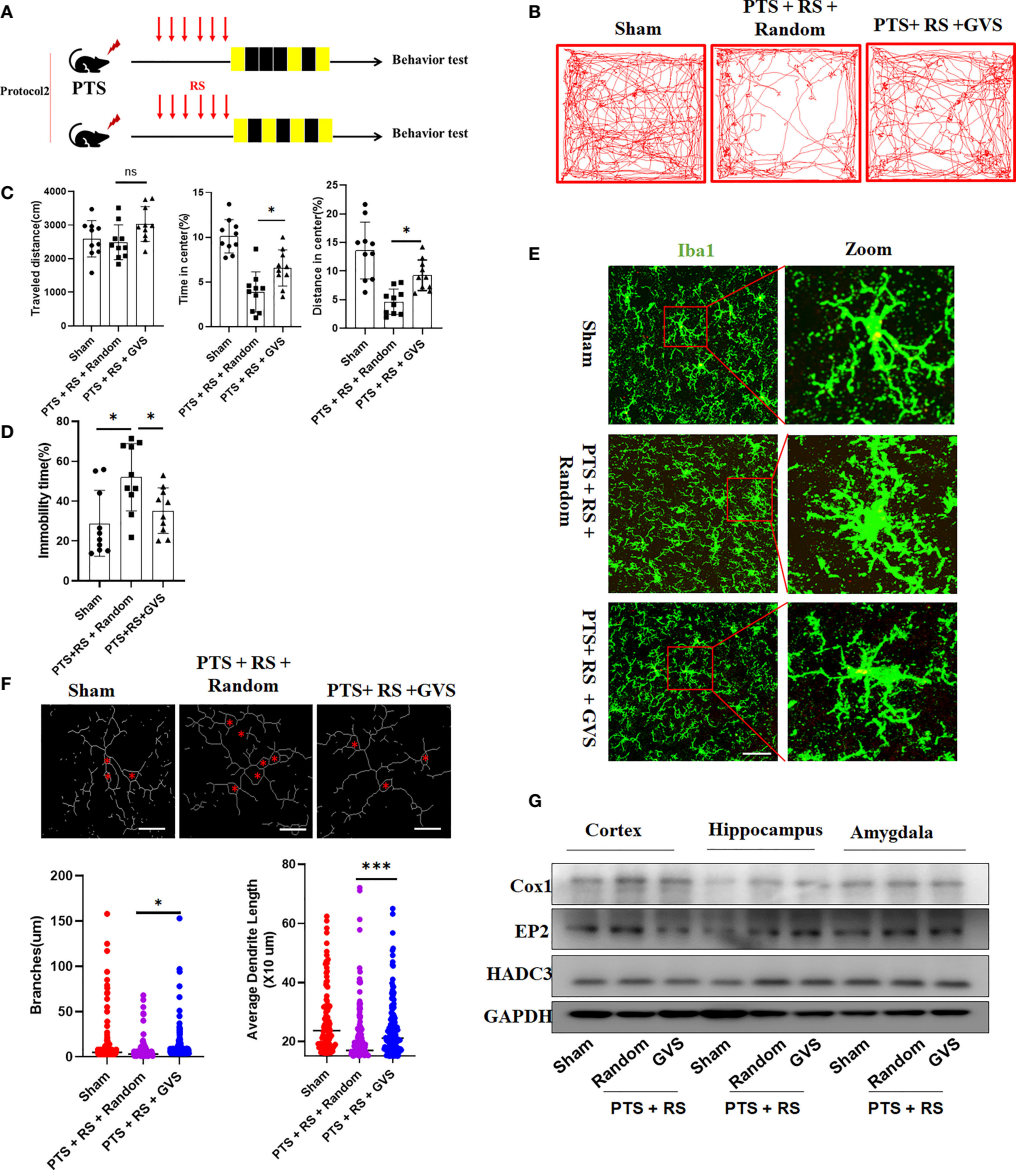
Gamma visual stimulation alleviates susceptibility to additional stress exposure after cortex infarction by modulating amygdala HDAC relative expression levels. **(A)** Timeline and design of the gamma visual stimulation experiments. **(B)** Representative tracks of open-field test (OFT) within the sham, PTS + RS + random and PTS + RS + GVS groups. **(C)** Statistical analysis of travel distance, percentage of distance travelled in center and time spent in center area within the three groups mentioned above (N = 10 per group). **(D)** Immobility time spent was tested by Tail suspension test (N = 10 per group). **(E)** Histological analysis of damaged cortex-nearly microglia. (Scale bar = 50um). **(F)** Statistical analysis of microglia morphology (ramification branches and dendrite length) from three various groups. **(G)** Western blot of HDAC3, Cox1 and EP2 in damaged cortex, hippocampus and amygdala in various group. The data mentioned above are presented as the means ± SEM and were analysed by one-way ANOVA with Bonferroni *post hoc* test (ns, no significance; *p < 0.05; ***p < 0.005).

The neuroimmune system, including glial cells and immune signalling molecules, offers a unique target to treat disease and improve brain health. However, little is known about how to nonpharmacologically manipulate the immune system of the brain. Gamma frequency (30-50 Hz), a component of the neuroimmune system, can be induced by sensory stimulation *via* LED light strips flickering at 40 Hz. Gamma visual stimulation has been reported to promote microglial activity, decrease amyloid-b (Ab) in Alzheimer’s disease, regulate mitochondrial fission in neurons and offer neuroprotection (28, 31–33). To explore whether gamma visual stimulation can alleviate anxiety susceptibility acquisition in PTS + RS, we designed and developed a device of gamma flicker stimulation such as the Tsai et al. description (33) (**Figures S11A, B**). Then, we found that gamma flicker stimulation can relieve anxious behaviour of PTS + RS as protocol 2 but not protocol 1 (**Figure 8A**, **Figure S11C**). Data shows that gamma flicker stimulation alleviates anxious or depression susceptibility to additional stress exposure after cortex infarction, with higher percentage of distance and time spent in center, lower immobility time spent (**Figures 8B–D** and **Figure S11C**). Next, to confirm whether gamma flicker stimulation improved synaptic function, we measured long-term potentiation (LTP) in hippocampal schaffer collaterals and observed a marked LTP trend towards better outcomes than random flicker stimulation (**Figure S11D**). In addition, microglial activation was suppressed, as shown in **Figures 8E, F**. However, the activation of astrocytes seemed to represent weak changes (**Figure S11E**). In addition, we investigated HDAC3/Cox/EP2 in PTS + RS with or without gamma flicker stimulation. Consequently, gamma flicker stimulation moderately decreases HDAC3 and Cox1 expression in the damaged cortex, weakly downregulated EP2 expression in amygdala (**Figure 8G**). Taken together, gamma visual stimulation alleviates susceptibility to additional stress exposure after cortex infarction by modulating HDAC3/Cox1/EP2 expression, mainly related to decrease pathway of PGE_2_ synthesis in damaged ischemic cortex.

## Discussion

In this study, by focusing on epigenetic remodelling factors reported to participate in anxiety susceptibility after stroke, we identified several findings that can explain molecular changes between nonstroke populations that generalize anxiety disorder and stroke patients with phobic anxiety. First, using an animal model assay to simulate two conditions of patients, we identified that PTS + RS manifests more severe anxious symptoms. Pathologically, microglial overactivation was widely observed in the PTS + RS group compared with the absolute restraint stress stimulus group. Inflammatory reaction disruption correlates with a negative affective state from rodents to humans (19, 34). Imaging studies and postmortem analysis indicate that microglial activation contributes to the development of negative affective emotion (15, 20). Therefore, evidence combined with previous theories and our results supported our hypothesis that microglia may be a key player in the progression of anxiety susceptibility. Next, RNA-seq enrichment provided insights that microglia were one of the most prominent factors, and immune-, phagosome-, ROS-generation pathways and ATP synthesis contribute closely to the phenotype of PTS + RS, further supporting the histological or molecular changes, as in previous reports (22).

Microglia have been proven to regulate mood by mediating inflammation signalling, but there is a lack of evidence to explore how microglia interact with the activity of neurons (21). Interleukin-6 (IL-6) and PGE_2_ are two direct messengers between microglia and neurons. IL-6 derived from activated microglia has been implicated in depression-like behaviour (35). During brain inflammation overactivation, metabolic disorders can lead to anxiety-like behaviour (36). Cox1 mediated cerebral PGE_2_ production involves anxiety sensitivity to environmental stress (37). In the PTS + RS group, the percentage of Cox1-enriched microglia was first reported to increase in the PTS + RS group, along with its downstream metabolite production. Although endothelial cells express Cox1, they have the highest expression in microglia (38). Cox1 is prominent in adverse effects at the early phase of inflammation, while Cox2-induced PGE_2_ production is critical for aversion symptoms in chronic inflammation conditions. In our case, we found that Cox1 and Cox2, downstream of NF-κB signalling, were upregulated constantly in the PTS + RS or RS group. In addition to PGE_2_ signalling participation, the Janus kinase (JAK)/signal transducer and activator of transcription (STAT) pathway is extremely activated, as our results show. Likewise, transcriptional elevations in the PGE_2_ synthesis enzymes Cox1 and Cox2, along with inflammatory factors such as IL-1β, TNF-α, IFN-γ, ASC and IL-6, are hallmarks of phenotype deterioration. These results demonstrate that PGE_2,_ as a common pathway metabolite, is highly enriched in microglia, eliciting anxiety susceptibility to additional stress exposure after cortex infarction. NF-κB-induced inflammatory reactions in the brain are closely associated with adverse affective phenotypes.

HDAC3 is a unique histone deacetylase of chromatin modifiers that silences transcription by interacting with nuclear receptor corepressors (NCoR1/2) (39). Deletion of HDAC3 in macrophages under LPS-induced systemic inflammatory reactions impairs the activation of proinflammatory target genes (11). Recent evidence has shown that HDAC3 is involved in I/R-induced brain injury and poststroke recovery and participates in neurodegenerative diseases such as Huntington’s disease or Parkinson’s disease (10). Whether HDAC3 contributes to the progression of phobic anxiety under stroke stress has not been reported. The HDAC3 inhibitor RGFP109 is an HDAC class I inhibitor selective for HDAC1 and HDAC3 and has been confirmed to be safe in phase I clinical trials in patients. However, the beneficial effects of RGFP109 are not reported in phobic anxiety. Notably, in this study, 60 mg/kg RGFP109 showed significant improvements, suggesting potential value in anxiety susceptibility. We found that NF-κB signalling was inhibited, along with inactivation of the STAT-inducible interferon pathway. In addition, we found that microglial synaptic pruning was greatly ameliorated. Simultaneously, glycogen synthase kinase-3 (GSK-3), a serine/threonine protein kinase existing as two isozymes, GSK-3 alpha (α) and GSK-3 beta (β), was analysed in our system for its roles in synapse plasticity (40). GSK-3 enzymatic activity is regulated dynamically; N-terminal phosphorylation of Ser21 for GSK-3α and Ser9 for GSK-3β exerts inhibitory regulation. Consequently, GSK-3 cannot access substrates (41). In contrast, tyrosine phosphorylation of Tyr279 in GSK-3α and Tyr216 in GSK-3β positively activated GSK-3. Chronic stress decreases synaptic density and causes synapse atrophy. High GSK-3β activity is required for pre- and postsynaptic molecular mechanisms to support the occurrence of LTD. Under our conditions, inhibitory regulation of Ser9 at GSK-3β was reduced during PTS + RS, while HDAC3 inhibition increased the expression levels of p-Ser9 GSK-3β and GSK-3β to approximate the sham group level, which maintained homeostatic mechanisms within synapses. Akt-mTOR signalling, one of the pathway phenotypes involved in the function of synapses by activating protein synthesis and alleviating depression or anxiety, is also enhanced upon HDAC3 inhibition.

Although HDAC3 inhibition positively alleviates inflammation by restraining NF-κB hyperactivation and regulates synaptic plasticity pathway phenotypes, such as GSK-3 and mTOR signalling, HDAC3 inhibition cannot solve the direct interaction between neuronal activity and activated microglia. NF-κB hyperactivation and stress susceptibility are both linked to nuclear p65 distribution. NF-κB-p65 can bind motifs by HDAC3 (42). First, we found that HDAC3 inhibition decreases damaged cortex PGE_2_ content by downregulating Cox1. EP2 expression in the amygdala is a core receptor region. Knockdown of EP2 in the amygdala significantly relieved anxiety-like behaviour in PTS + RS. Therefore, an HDAC3 remoulded Cox1-PGE_2_-EP2 network was established. Furthermore, we found that Cox1, as the target gene downstream of NF-κB signalling, depends on HDAC3 regulation. As proven by a previous report, HDAC3 contains deacetylase (DA)-dependent deacetylation of p65, in line with previous literature indicating that p65 is a direct substrate of HDAC3 and that deacetylation triggers the nuclear export of p65 (43, 44). Therefore, HDAC3 regulating PGE_2_ synthesis may be attributed to deacetylating p65, as our data proved. Therefore, in phase summary, our findings reveal the HDAC3/Cox1/EP2 signalling axis in susceptibility to poststroke anxiety.

To explore a new treatment strategy, gamma visual stimulation is a candidate approach to reduce PTS + RS pathology. Gamma oscillation (30-90 Hz) is closely associated with cognitive functions, Alzheimer’s disease, stroke recovery and emotion regulation (32). According to a previous report, non-invasive stimulation by specific frequency flickering light could evoke gamma oscillation. A recent study provided evidence of low gamma oscillation in free-behaving mice in cerebral ischaemia, and light flickers can restore low gamma to confer neuroprotection (31, 45). However, whether gamma visual stimulation can alleviate anxiety-like behaviour in PTS + RS has not been validated. Here, using gamma entrainment *via* visual stimuli, we also observed that the HDAC3/Cox1/EP2 signalling axis was changed, alongside significant reductions in activated microglia. Gamma flicker stimulation moderately decreased HDAC3 and Cox1 expression in the damaged cortex and weakly downregulated EP2 expression in the amygdala.

## Conclusions

In summary, we identified the role of the HDAC3/Cox1/EP2 signalling network in susceptibility to poststroke anxiety, and a protective approach of gamma visual stimulation might be a candidate strategy for alleviating susceptibility to additional stress exposure after cortex infarction. Further investigations on how HDAC3 is regulated or modified during anxiety-like behaviour and whether there are other messengers travelling through within-brain regions to affect neuron activity may accelerate the discovery for a long period.

## Data Availability Statement

The data presented in the study are deposited in the GEO repository, accession number: GSE194326.

## Ethics Statement

The animal study was reviewed and approved by The University Committee on Animal Care of Xiamen University.

## Author Contributions

HZ designed the project. HZ, YG, and BD contributed to the analysis of data and drafting of the manuscript. HZ, YG, AH, HS, YC, JS, AG, HW, and JS conducted all experiments. HZ and BD participated in discussions, data analysis, and manuscript editing. All authors have read and approved the final version of the manuscript.

## Funding

This work was partly supported by the National Natural Science Foundation of China (81501207), the General project of Fujian Natural Science Foundation (No. 2021J01018), the Projects of the principal fund of Xiamen University (No. 20720210118), the Science and Technology Project of Xiamen municipal Bureau of Science and Technology (3502Z20194046), the Fundamental Research Funds for the Central Universities (20720200037), Key scientific research projects of Fujian Strait medical and Health Exchange Association (2020HYH10), Fujian Health Science and technology program (2021GGB038), XMU Undergraduate Innovation and Entrepreneurship Training Programs (2021X1073; S202110384894).

## Conflict of Interest

The authors declare that the research was conducted in the absence of any commercial or financial relationships that could be construed as a potential conflict of interest.

## Publisher’s Note

All claims expressed in this article are solely those of the authors and do not necessarily represent those of their affiliated organizations, or those of the publisher, the editors and the reviewers. Any product that may be evaluated in this article, or claim that may be made by its manufacturer, is not guaranteed or endorsed by the publisher.
