# Supplementary material for: HDAC3-Regulated PGE2 Production by Microglia Induces Phobic Anxiety Susceptibility After Stroke and Pointedly Exploiting a Signal-Targeted Gamma Visual Stimulation New Therapy
Source: Front Immunol. 2022 Feb 18;13:845678. doi: 10.3389/fimmu.2022.845678 (PMC8895955; doi:10.3389/fimmu.2022.845678)
Supplement: Supplementary file 1 [file DataSheet_1.docx]

**Supplemental Information**

**HDAC3-regulated PGE2 production by microglia induces phobic anxiety susceptibility after stroke and pointedly exploiting a signal-targeted gamma visual stimulation new therapy**

Figure S1. The acquisition of anxiety-like behaviours after cortex infarction is independent of amygdala organic damage.

Figure S2. Restraint stress exposure after cortex infarction increases the degree of susceptibility to adverse experiences.

Figure S3. Restraint stress exposure after cortex infarction exacerbates hyperreactivity of the amygdala.

Figure S4. Injury-related differential genes and pathway changes between restraint stress and photothrombotic stroke plus restraint stress mice.

Figure S5. Time series of relative inflammatory gene changes between restraint stress and photothrombotic stroke plus restraint stress mice.

Figure S6. HDAC3 inhibition alleviates anxiety-like behaviour and synaptic damage.

Figure S7. Anxiety-like behaviour is dependent on EP2 blockade after additional stress exposure plus cortical infarction.

Figure S8. Integrated analysis of downstream target pathways regulated by HDAC3.

Figure S9. Excessive inflammation in the brain of PTS + RS can be suppressed by HDAC3.

Figure S10. Effect of HDAC3 gene interference and its interacting molecules.

Figure S11. Gamma visual stimulator introduction and intervention program.

**Figure S1.** **The acquisition of anxiety-like behaviours after cortex infarction is independent of amygdala organic damage.**

**
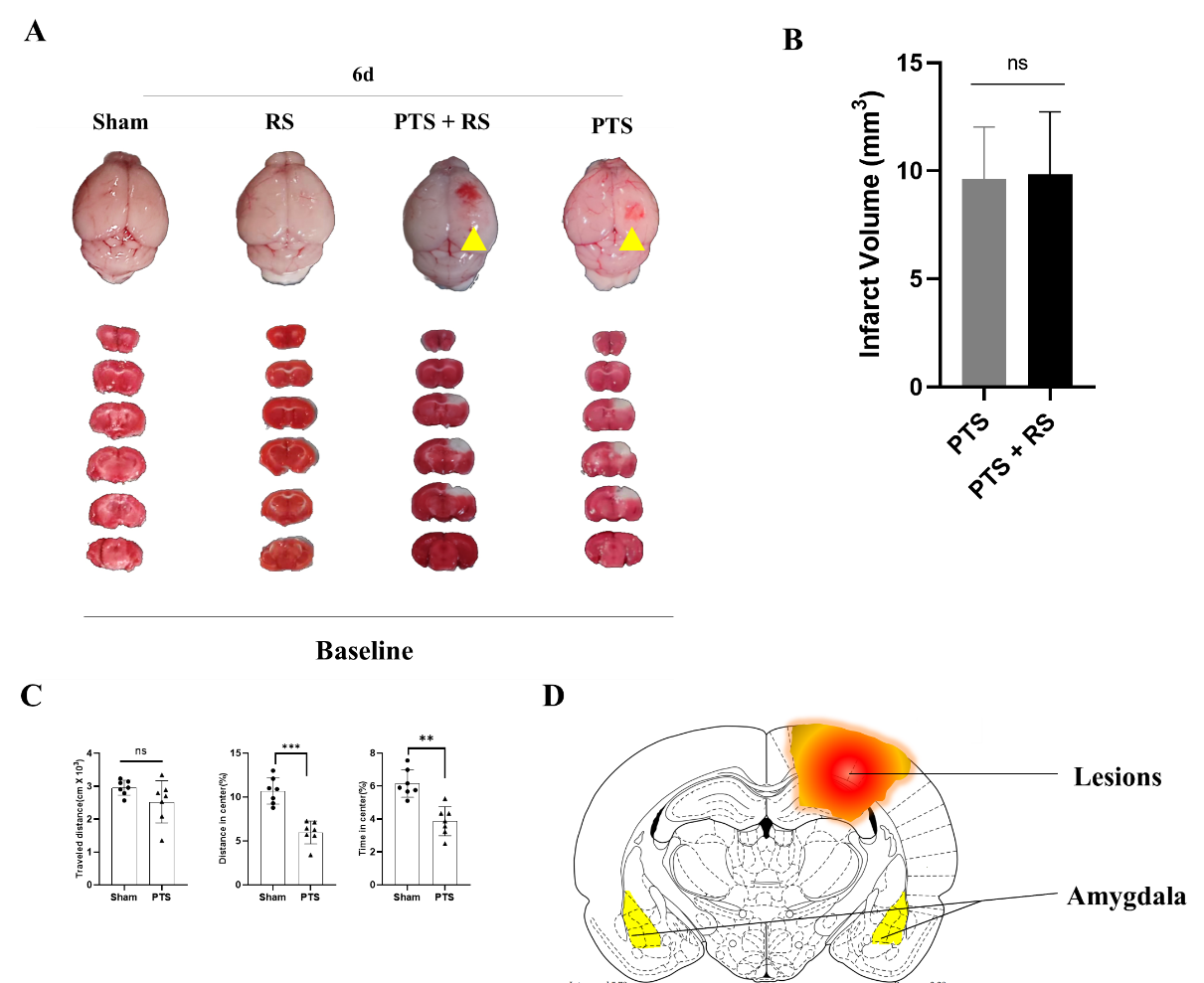
**

A. General observation and TTC staining of the brains of the sham, RS, PTS and PTS + RS groups. (Yellow arrow reflects the infarction lesion in the cortex). B. Statistical analysis of infarct volume of PTS and PTS + RS. C. Distance travelled in the centre (%) and time travelled in the centre (%) of the sham and PTS groups on the fourth day of the acute phase after modelling. D. Infarction lesion area illustrated with red and amygdala far from lesions marked with yellow. This brief graph reflects that the activation of the amygdala involved in anxiety-like behaviours is independent of organic damage after cortex infarction. The data mentioned above are presented as the means ± SEM and were analysed by Student’s t-test when comparing 2 groups. (. ns, no significance; *p < 0.05; **p < 0.01; ***p < 0.005.).

**Figure S2.** **Restraint stress exposure after cortex infarction increases the degree of susceptibility to adverse experiences.**


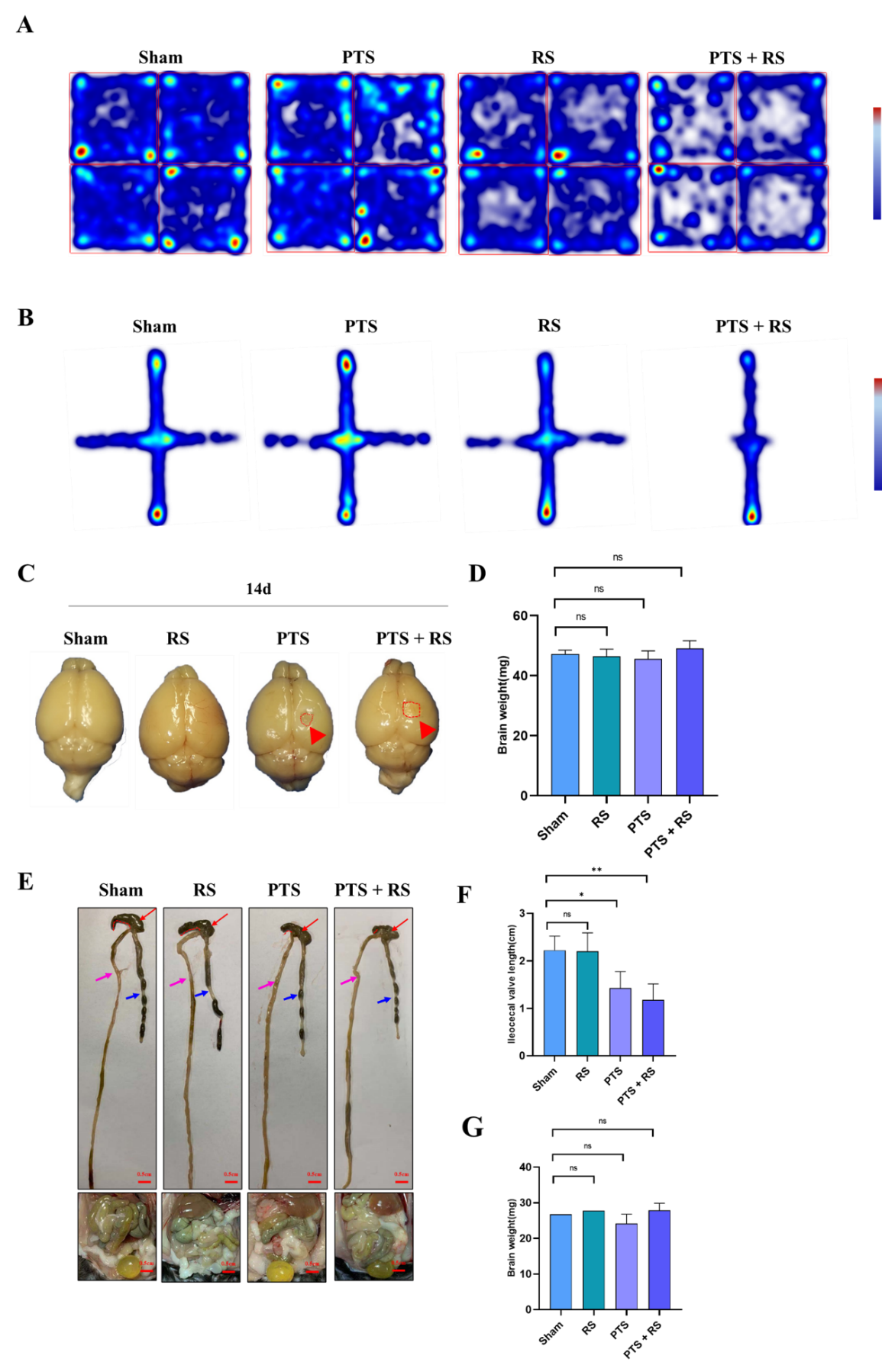


A. Open-field test on day 31 after inducing model. Track hot map of sham, PTS, RS and PTS + RS. B. Track hot map of elevated plus-maze in various groups. C~D. C: General observation of the damaged cortex of the sham, PTS, RS and PTS + RS groups (the red arrow shows the liquefaction cavity in the subacute stage). D: Brain weight of various groups on Day 14. E~F. General observation of internal organs of the abdominal cavity, small intestine, caecum and rectum (E, scale bar = 0.5 cm) G. Brain weight of various groups on the last day of behaviour tests. The data mentioned above are presented as the means ± SEM and were analysed by one-way ANOVA with Bonferroni post hoc test (. ns, no significance; *p < 0.05; **p < 0.01; ***p < 0.005.).

**Figure S3. Restraint stress exposure after cortex infarction exacerbates hyperreactivity of the amygdala.**


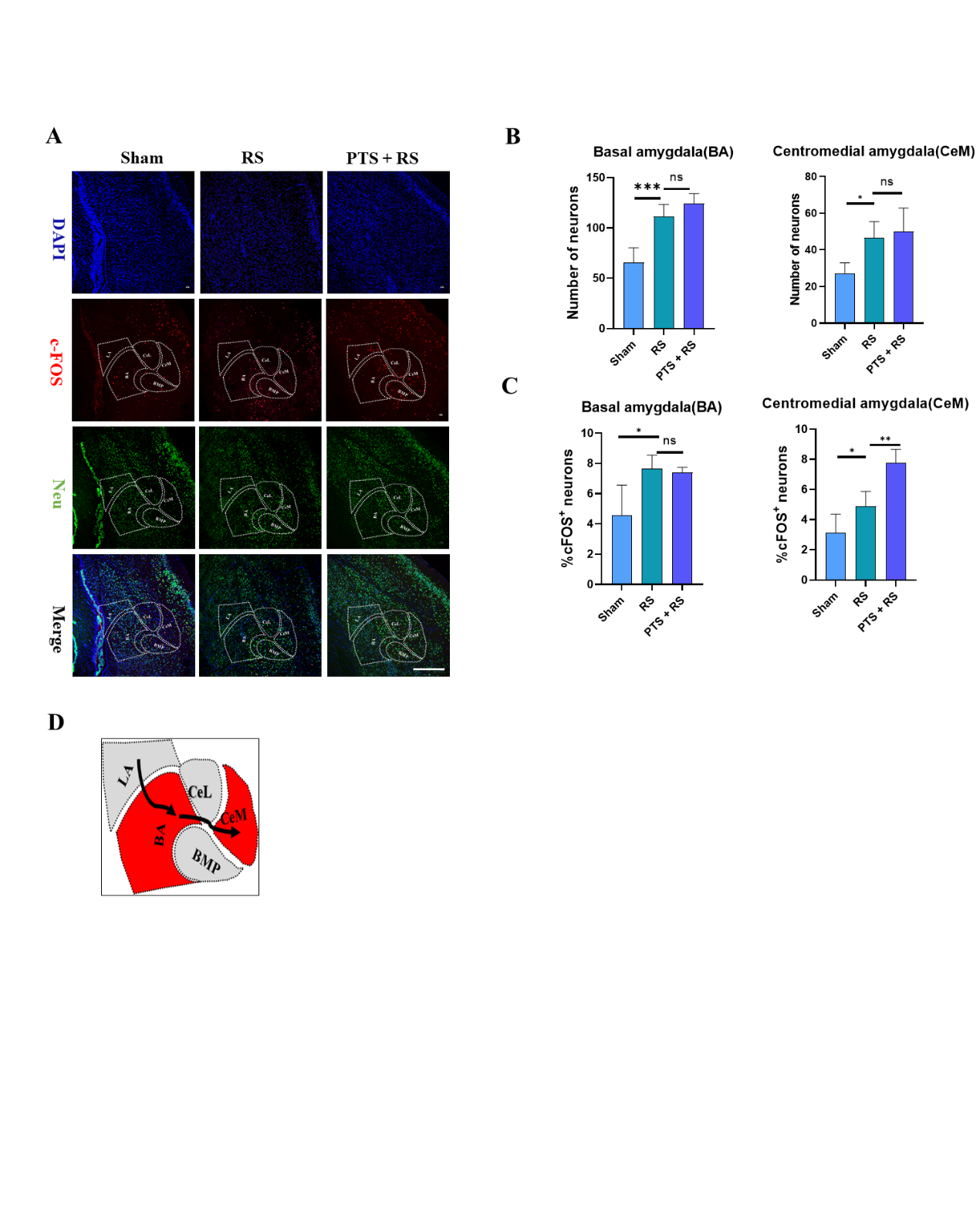


A. Immunofluorescence of c-Fos and NeuN in the amygdala and its subareas (lateral amygdala (LA), basal amygdala (BA), central amygdala (CeL), centromedial amygdala (CeM) and posterior basal amygdala within Sham, RS and PTS + RS groups. B~C. Statistical analysis of the number of neurons and c-Fos neurons in corresponding nuclear areas (BA and CeM). D. Amygdala anxiety circuitry. BA neurons project to the centrolateral (CeL) and CeM nuclei. The CeM represents the primary output nucleus of the amygdala, which projects directly to downstream targets to mediate anxiety-related behaviours. The data mentioned above are presented as the means ± SEM and were analysed by one-way ANOVA with Bonferroni post hoc test (. ns, no significance; *p < 0.05; **p < 0.01; ***p < 0.005.).

**Figure S4. Injury-related differential genes and pathway changes between restraint stress and photothrombotic stroke plus restraint stress mice.**


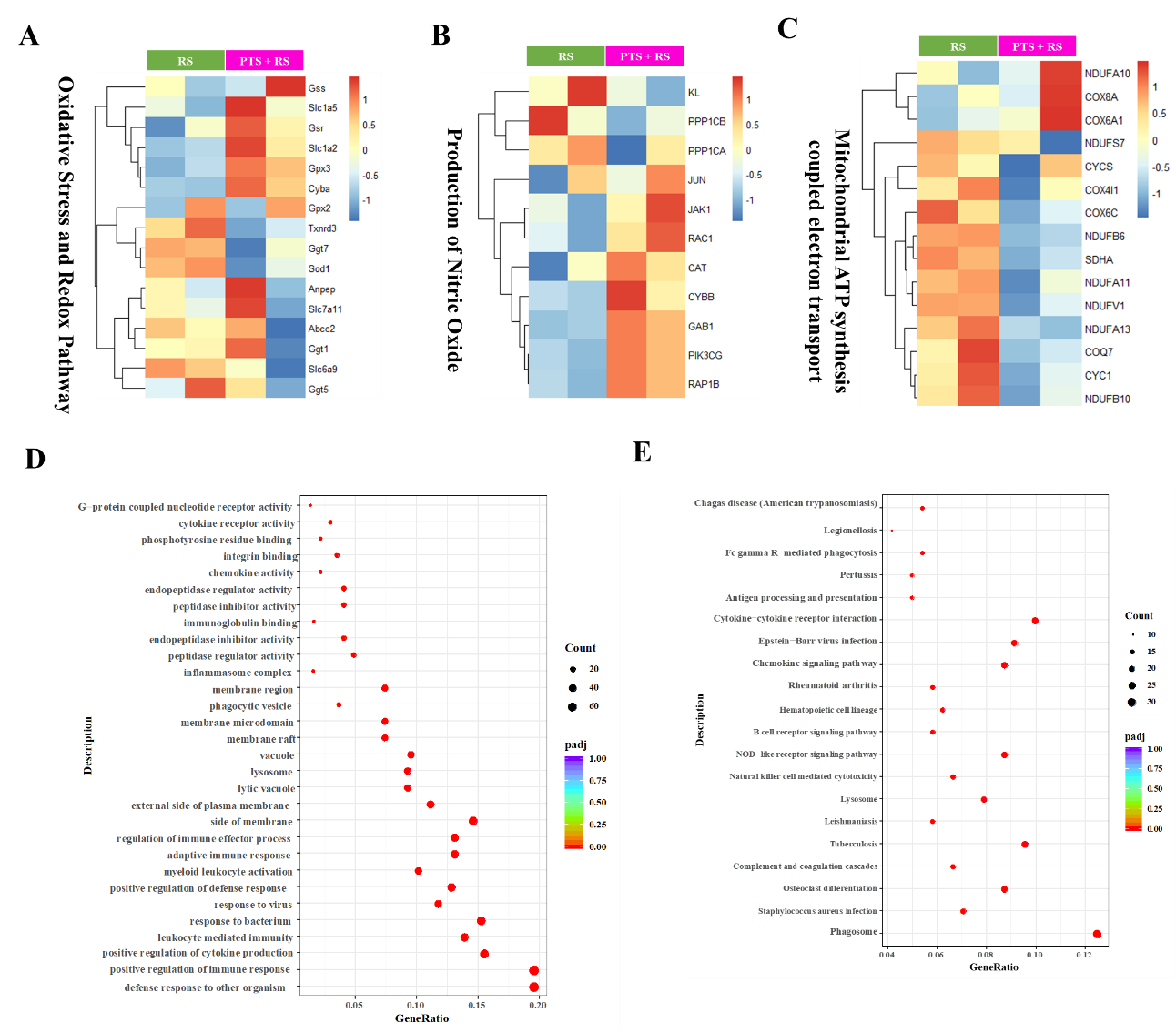


A. Oxidative stress- and redox pathway-related gene map related different genes between PTS + RS and RS group based on the RNA-seq dataset on the day of 21. B. Heatmap showing contents of production of nitric oxide pathway relative genes between PTS + RS and RS group based on RNA-seq analysis. C. Mitochondrial ATP synthesis-coupled electron transport-related genes between PTS + RS and RS group based on RNA-seq analysis. D. GO (Gene Ontology, upregulated enrichment) analysis between two groups based on RNA-seq analysis. E. KEGG (Kyoto Encyclopedia of Genes and Genomes) analysis of upregulated pathways between the two groups based on RNA-seq analysis. For detail information please refer to Supplementary Table 2.

**Figure S5. Time series of relative inflammatory gene changes between restraint stress and photothrombotic stroke plus restraint stress mice.**


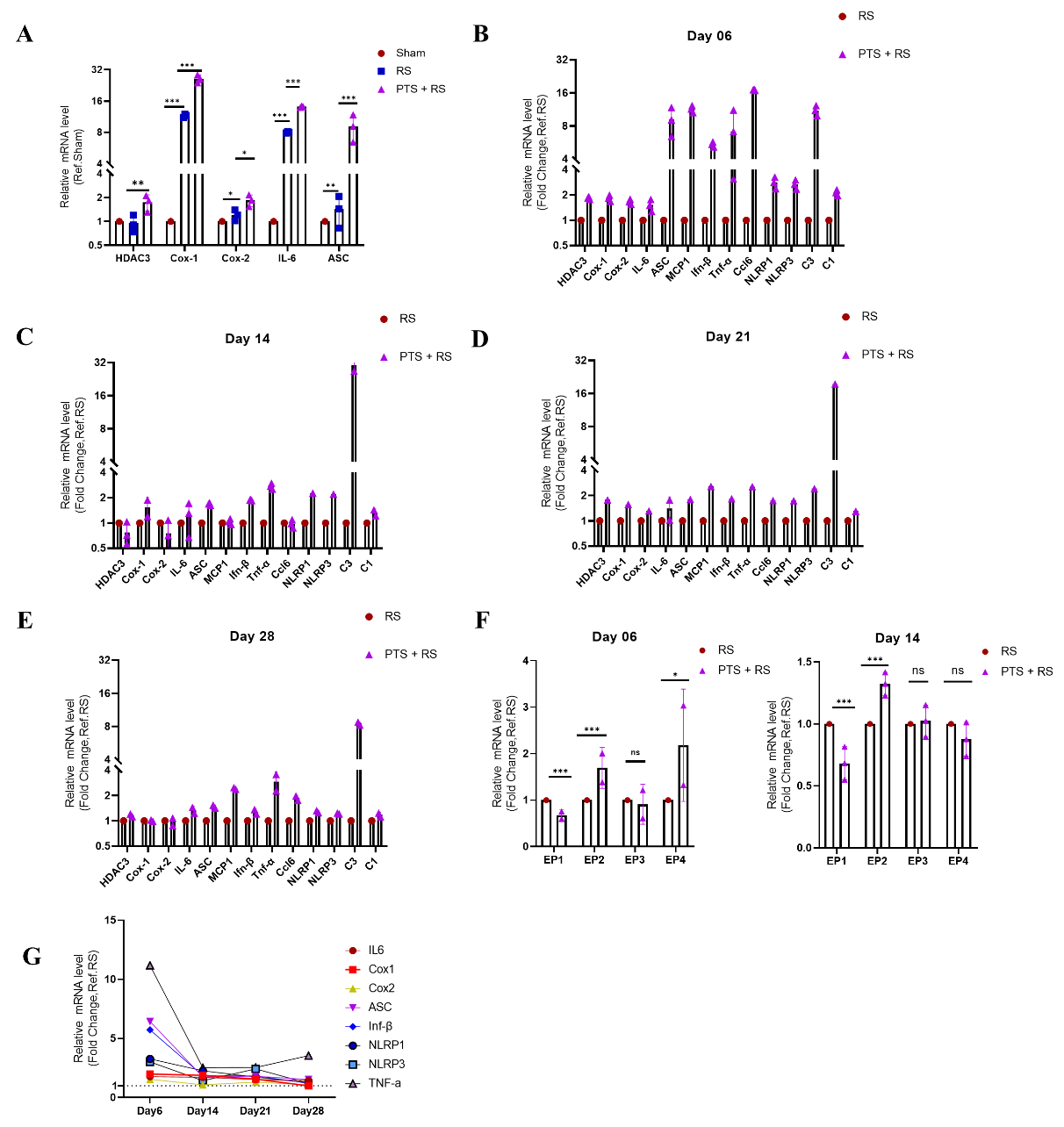


A. General different gene changes reflected by qRT-PCR between sham, PTS + RS and RS on the day of 21. B~ E. qRT-PCR outcomes of different genes between PTS + RS and RS on days 6, 14 and 28. F. Prostaglandin receptor (EP1, EP2, EP3 and EP4) changes in the acute stage on day 6 and subacute phase on day 14 between PTS + RS and RS group. The data mentioned above are presented as the means ± SEM and were analysed by one-way ANOVA with Bonferroni post hoc test or Student’s t-test when comparing 2 groups. (. ns, no significance; *p < 0.05; **p < 0.01; ***p < 0.005.).

**Figure S6. HDAC3 inhibition alleviates anxiety-like behaviour and synaptic damage.**


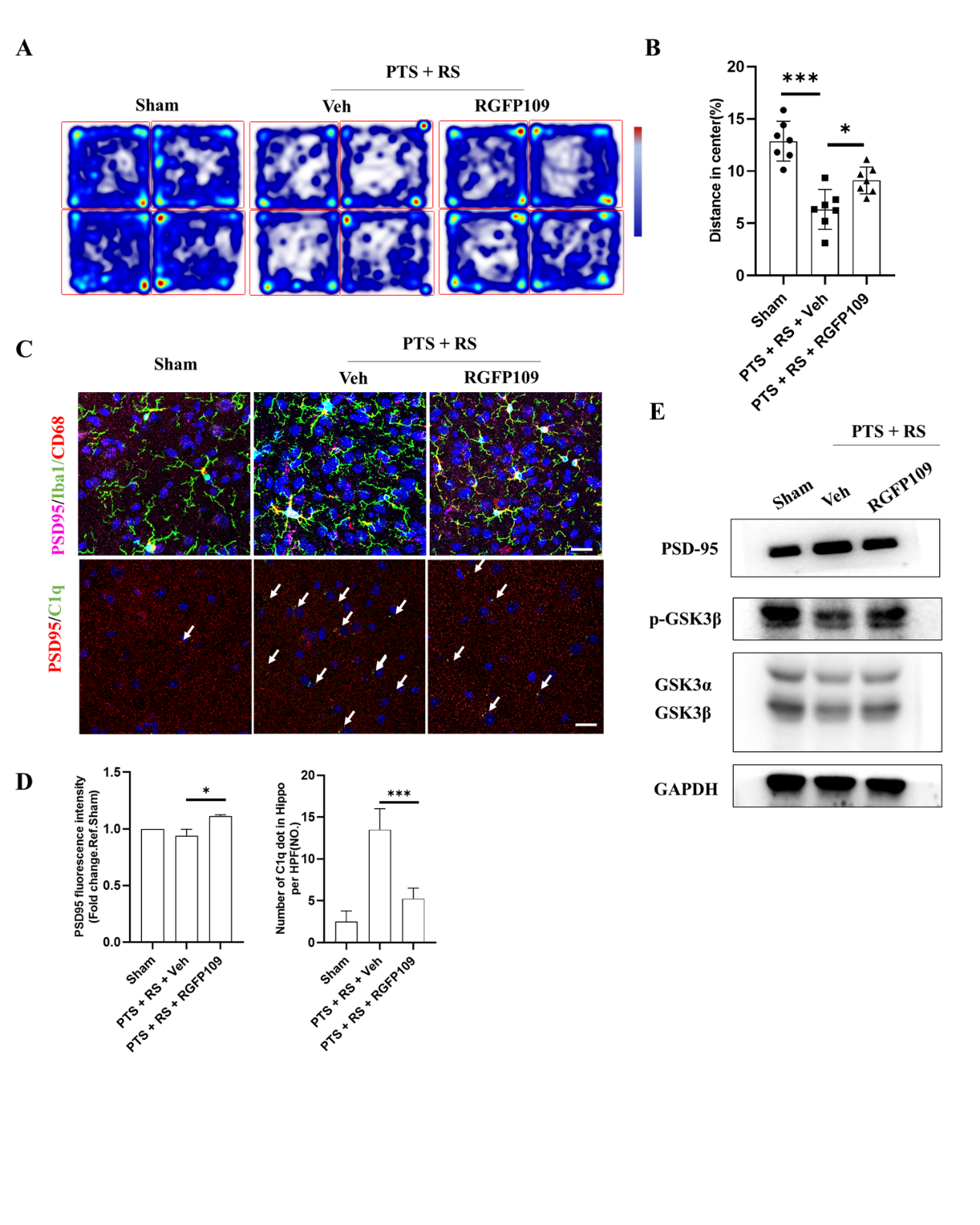


A. Open-field test. Track hot map of sham as a negative control, vehicle and RGFP109 of PTS + RS. B. Statistical analysis of distance in centre (%) of three groups. C. Immunofluorescence of PSD95, CD68, Iba1 and C1q in damaged cortex in sham, vehicle and RGFP109. D. Statistical analysis of PSD95 fluorescence intensity and number of C1q dots in the hippocampus per high-power field (HPF). E. Western blot of PSD95, pSer9-GSK3β, GSK3α, GSK3β and GAPDH in the three groups. The data mentioned above are presented as the means ± SEM and were analysed by one-way ANOVA with Bonferroni post hoc test or Student’s t-test when comparing 2 groups. (. ns, no significance; *p < 0.05; **p < 0.01; ***p < 0.005.).

**Figure S7. Anxiety-like behaviour is dependent on EP2 blockade after additional stress exposure plus cortical infarction**


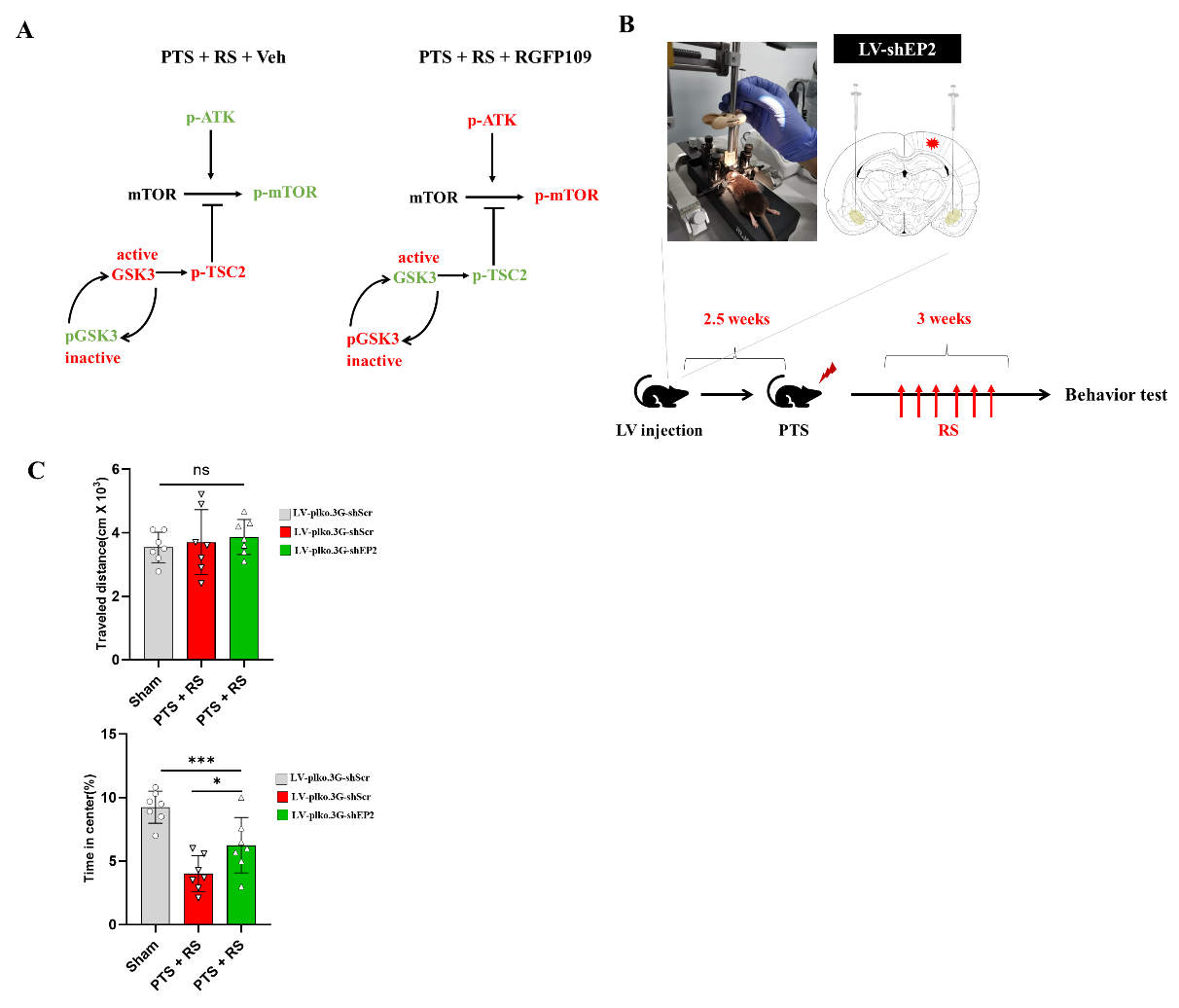


A. Biological effect of HDAC3 inhibitor represents the mechanism model diagram. B. shEP2 lentivirus interference of the bilateral amygdala for 3 weeks. C. Statistical analysis of travelled distance and time spent percentage in centre in PTS + RS with or without EP2 knockdown. The data mentioned above are presented as the means ± SEM and were analysed by one-way ANOVA with Bonferroni post hoc test or Student’s t-test when comparing 2 groups. (. ns, no significance; *p < 0.05; **p < 0.01; ***p < 0.005.).

**Figure S8. Integrated analysis of downstream target pathways regulated by HDAC3.**


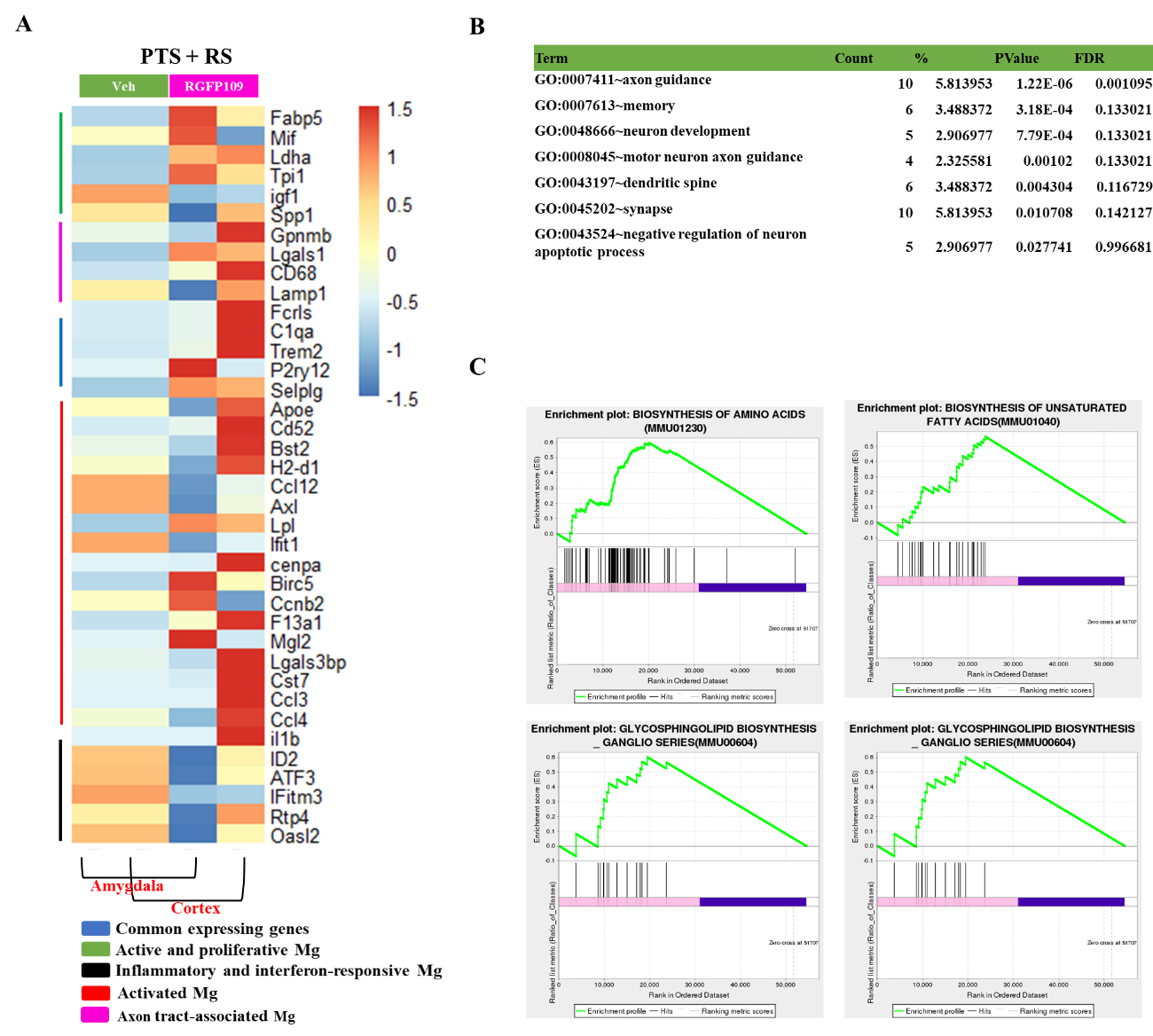


A. Unique microglial gene changes between PTS + RS with or without HDAC3 inhibition. B~ C. Bioinformatics analysis of GO (up enrichment) and GSEA pathways between vehicle and RGFP109 groups. For detail information please refer to Supplementary Table 3.

**Figure S9. Excessive inflammation in the brain of PTS + RS can be suppressed by HDAC3.**


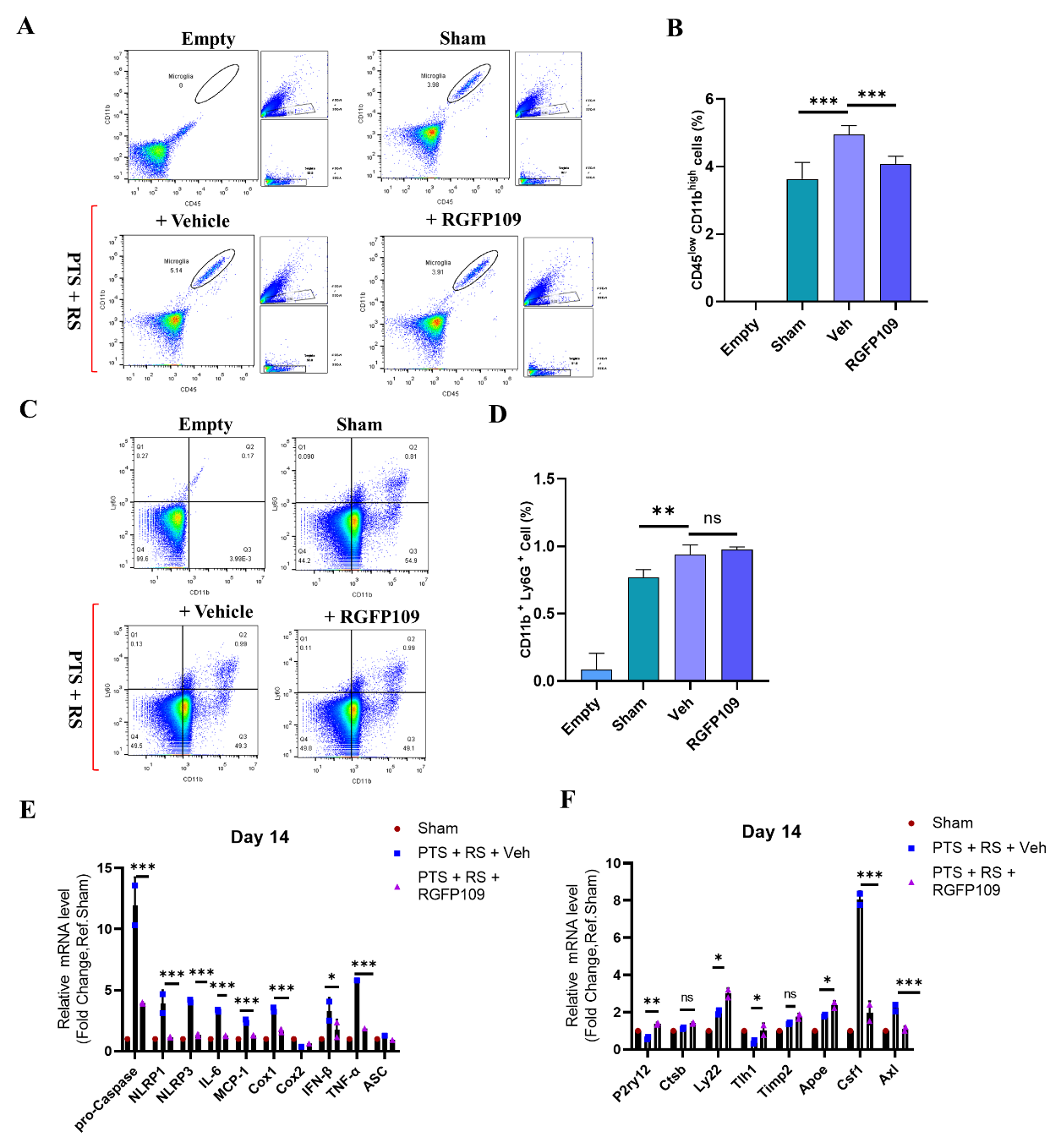


A~B. Flow cytometric analysis of microglia (CD45^low^ CD11b^high^) percentage in empty mice for the negative control, sham, vehicle and RGFP109 groups and corresponding statistical outcomes. C. Flow cytometric analysis of neutrophils (CD11b^+^Ly6G^+^) in the three groups and the corresponding statistical outcomes. E. qRT-PCR outcomes of relative inflammatory factors within the sham, vehicle and RGFP109 PTS + RS groups. F. Disease-associated microglia unique marker genes within the sham, vehicle and RGFP109 + PTS + RS groups. The data mentioned above are presented as the means ± SEM and were analysed by one-way ANOVA with Bonferroni post hoc test or Student’s t-test when comparing 2 groups. (. ns, no significance; *p < 0.05; **p < 0.01; ***p < 0.005.).

**Figure S10. Effect of HDAC3 gene interference and its interacting molecules.**


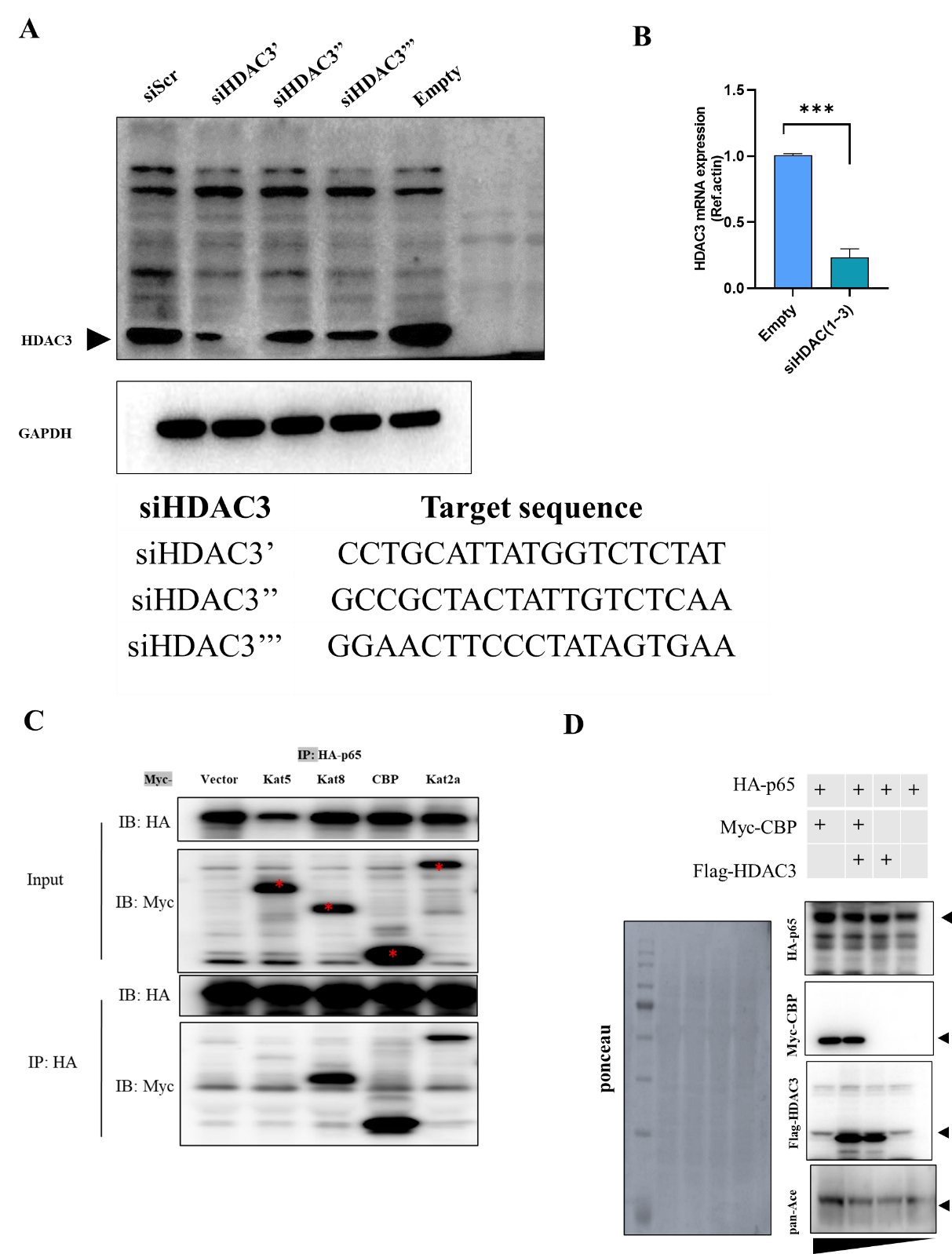


A. Western blots assay: the efficiency of siHDAC3s for the treatment of primary microglia for 48h.B. Q-PCR outcomes of siHDAC3s interference efficiency. C. Co-IP assay of HA-p65 and other acetyltransferases. D. Total acetylation modification detection of HA-p65, overexpressing myc-CBP in HEK293T cells with or without Flag-HDAC3. Data mentioned above represented as means ± SEM and analysed by Student’s t test when comparison of 2 groups (. ns, no significance; *p < 0.05; **p < 0.01; ***p < 0.005.).

**Figure S11. Gamma visual stimulator introduction and intervention program.**

**
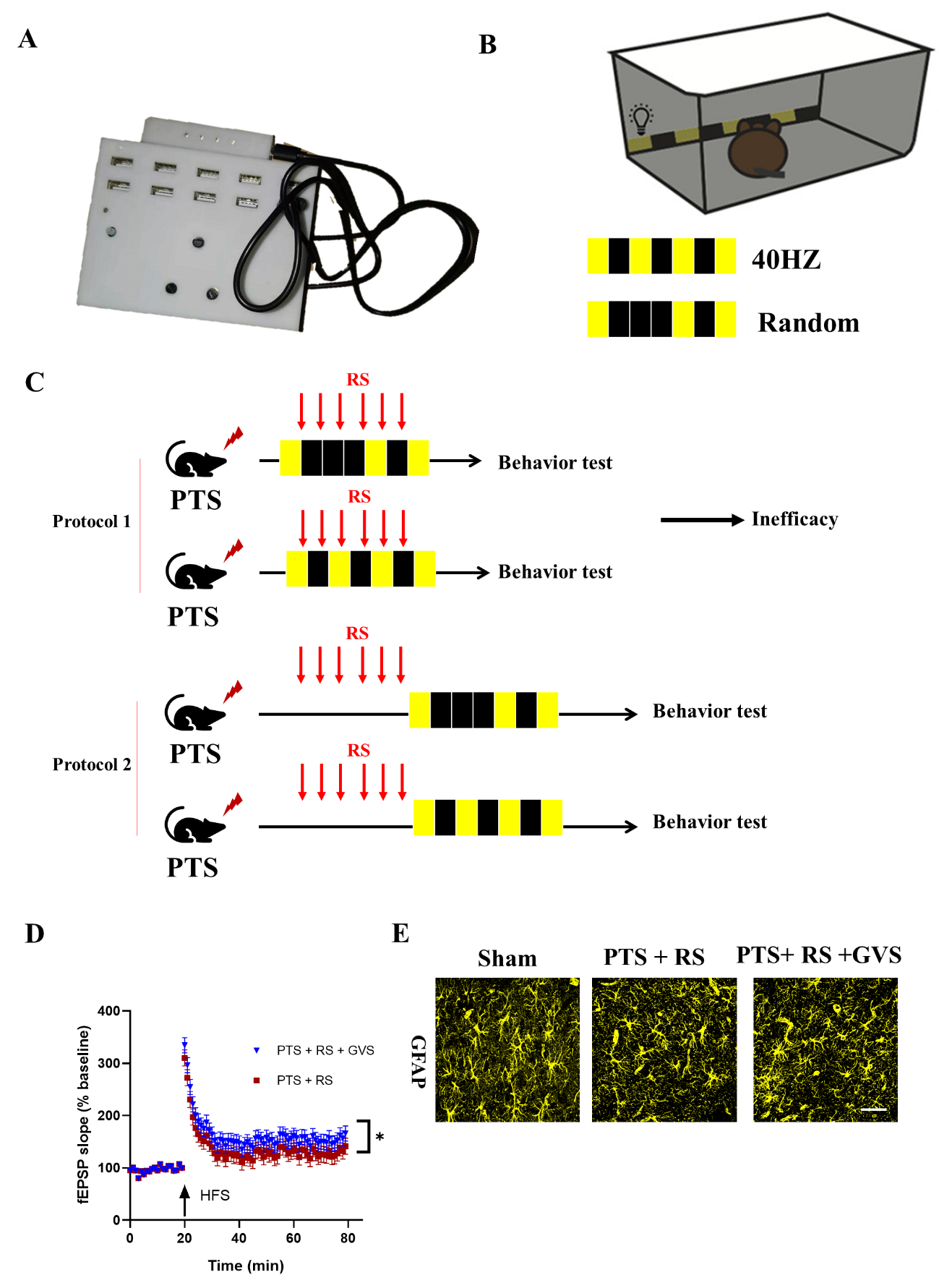
**

A. Example device of gamma flicker stimulation. B. Experimental configuration for presenting visual stimulation. C. 40 Hz visual flicker stimulation protocol. 40 Hz (12.5 ms light on, 12.5 ms light off), or random (light pulses were delivered with a random interval) stimulation for 2 h one day. Protocol No. 1: Mice underwent PTS; RS was fulfilled on the 7^th^ day every day at 9 am~11 am for 21 days, and gamma oscillation (or random visual stimulation) treatment was managed at 18.00 pm~ 20.00 pm simultaneously after the acute period passed. Protocol No. 2: Mice underwent PTS first, and then RS was fulfilled on the 7^th^ day every day at 9 am~11 am for 21 days. Gamma oscillation treatment (or random visual stimulation) was managed at 18.00 pm~ 20.00 pm every day for three weeks after PTS + RS intervention was finished. D. Electrophysiologic detection of LTP on Day 21 between the two groups (N = 3/groups). E. GFAP staining between three groups for astrocyte imaging (Scale bar = 50 µm).
